# Understanding radiation response and cell cycle variation in brain tumour cells using Raman spectroscopy†

**DOI:** 10.1039/d3an00121k

**Published:** 2023-05-01

**Authors:** Iona E. Hill, Marie Boyd, Kirsty Milligan, Cerys A. Jenkins, Annette Sorensen, Andrew Jirasek, Duncan Graham, Karen Faulds

**Affiliations:** a Centre for Molecular Nanometrology, Department of Pure and Applied Chemistry, Technology and Innovation Centre, University of Strathclyde 99 George Street Glasgow G1 1RD UK karen.faulds@strath.ac.uk; b Strathclyde Institute of Pharmacy and Biomedical Sciences, University of Strathclyde Glasgow G1 1XQ UK; c Department of Physics, The University of British Columbia Kelowna Canada; d Swansea University Medical School, Swansea University Singleton Park Swansea SA2 8PP UK

## Abstract

Radiation therapy is currently utilised in the treatment of approximately 50% of cancer patients. A move towards patient tailored radiation therapy would help to improve the treatment outcome for patients as the inter-patient and intra-patient heterogeneity of cancer leads to large differences in treatment responses. In radiation therapy, a typical treatment outcome is cell cycle arrest which leads to cell cycle synchronisation. As treatment is typically given over multiple fractions it is important to understand how variation in the cell cycle can affect treatment response. Raman spectroscopy has previously been assessed as a method for monitoring radiation response in cancer cells and has shown promise in detecting the subtle biochemical changes following radiation exposure. This study evaluated Raman spectroscopy as a potential tool for monitoring cellular response to radiation in synchronised *versus* unsynchronised UVW human glioma cells *in vitro*. Specifically, it was hypothesised that the UVW cells would demonstrate a greater radiation resistance if the cell cycle phase of the cells was synchronised to the G_1_/S boundary prior to radiation exposure. Here we evaluated whether Raman spectroscopy, combined with cell cycle analysis and DNA damage and repair analysis (γ-H2AX assay), could discriminate the subtle cellular changes associated with radiation response. Raman spectroscopy combined with principal component analysis (PCA) was able to show the changes in radiation response over 24 hours following radiation exposure. Spectral changes were assigned to variations in protein, specifically changes in protein signals from amides as well as changes in lipid expression. A different response was observed between cells synchronised in the cell cycle and unsynchronised cells. After 24 hours following irradiation, the unsynchronised cells showed greater spectral changes compared to the synchronised cells demonstrating that the cell cycle plays an important role in the radiation resistance or sensitivity of the UVW cells, and that radiation resistance could be induced by controlling the cell cycle. One of the main aims of cancer treatment is to stop the proliferation of cells by controlling or halting progression through the cell cycle, thereby highlighting the importance of controlling the cell cycle when studying the effects of cancer treatments such as radiation therapy. Raman spectroscopy has been shown to be a useful tool for evaluating the changes in radiation response when the cell cycle phase is controlled and therefore highlighting its potential for assessing radiation response and resistance.

## Abstract 2

The use of Raman spectroscopy to determine the radiation response of human glioma cells and how this response differs depending on the cell's position in the cell cycle.

## Graphical abstract

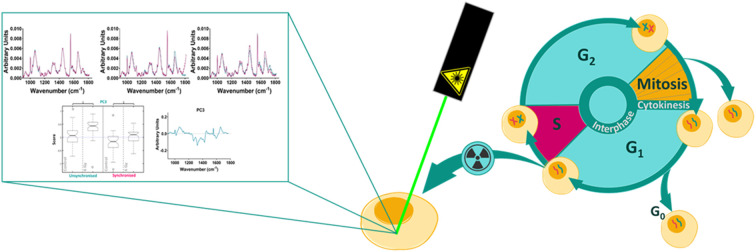

Every year in the UK, there are over 12 000 new brain, central nervous system and intracranial tumour cases diagnosed making brain tumours the 9^th^ most common type of cancer.^1^ These tumours account for over 5000 deaths annually in the UK and the ten-year survival rate for brain tumours is only around 15%, which is one of the lowest of all types of cancer.^1,2^ Difficulty treating cancers in the brain accounts for the poor patient survival.

For brain tumours, surgery is most commonly used to remove the bulk of the tumour, however surgery is not always possible due to the location of the tumour and delicate nature of the brain.^3^ For this reason, surgery is often used in combination with radiation therapy and chemotherapy, in particular temozolomide treatment.^4^ Radiation therapy is common across all types of cancer with over 50% of cancer patients receiving radiotherapy as part of their treatment regime.^3,5^ In an organ like the brain, damage to healthy tissue surrounding the tumour must be minimised, as damage to healthy brain tissue could have detrimental effects for the patients’ cognitive function. Therefore, the dose used in radiation therapy must be kept as low as possible by utilising conformal radiation therapy and extended treatment times. The total clinical dose of radiation administered is normally between 45 and 60 Gy, however, a patient will receive regular lower doses (1.5–3 Gy) over a long period of time to make up this total dose.^3^ This method of treatment is known as radiation fractionation, where dividing the overall dose into smaller doses reduces the toxic effects to healthy cells.^6^

The treatment limitations of brain tumours have led to a drive towards patient tailored radiation therapy. Specifically, a range of innovations have been developed to adapt the treatment regime to a patient's specific needs, including methods of predicting radiation resistance,^7–9^ ways to monitor radiation response in real time^10^ or the use of radiosensitising agents.^11–14^ However, currently there are no clinically-implemented methods for assessing tumour radiation response in patients during the course of their treatment. Radiation response and the radiobiology of tumours and tissue is normally assessed *in vitro* in cancer cell lines and 3D tumour models assessing cell survival,^15^ apoptosis,^16^ hypoxia,^17,18^ deoxyribonucleic acid (DNA) damage and repair^19,20^ and cell cycle arrest.^21^ These methods are often time-consuming and destructive to the sample and therefore pose difficulties for translation to the clinic. Due to the heterogeneous nature of tumours between, and within patients, there is significant variability between how patients respond to therapy. This limits the efficacy of treatment, as lower doses of therapy are given to reduce the toxicity to the most sensitive within the population. Thus, understanding the differences in patient response would aid in improving patient tailored therapy and combination therapies.

Raman spectroscopy has previously been evaluated as a tool for monitoring radiation response at a cellular level.^22–31^ Raman is a vibrational spectroscopic technique which is non-invasive and non-destructive therefore allowing analysis of cell and tissue samples while maintaining integrity of the sample.^32,33^ This technique can be used to gain information about the molecular composition of a sample and to evaluate subtle biochemical changes at a sub-cellular level. The breadth of information that can be gained makes Raman spectroscopy a promising method to use for assessing metabolic changes in cancer cells at both the cellular and molecular level when exposed to a stressor, such as ionising radiation.

Raman spectroscopy has successfully been applied to the study of radiation response in both single cells^22–31^ and tumour models.^34–37^ Early studies by Matthews *et al.* investigated the radiation response of prostate, breast, and lung cancer cell lines when irradiated with 15–50 Gy.^22,23^ Over 72 hours following irradiation, spectral changes arising from changes in cellular concentrations of amino acids, conformational protein structures, nucleic acids and lipid groups were evaluated using principal component analysis. These changes were proposed to be radiation response mechanisms associated with the synthesis and degradation of structured proteins and the expression of anti-apoptotic factors or other survival signals. Later studies confirmed these findings at clinically relevant doses.^24,34^ In addition, a study by Van Nest *et al.* used Raman spectroscopy to demonstrate cellular response was observed as early as 2 hours following 15 Gy irradiation and Raman detectable differences remained even 10 days after treatment.^36^

Previous studies have also shown that Raman spectroscopy could be used to measure changes to cellular glycogen levels that occurred following exposure to X-ray radiation.^24,30,31,34,36^ These studies demonstrated a radiation dose dependent accumulation of glycogen in cancer cells following X-ray irradiation. However, this response was dependent on tumour origin, sample type and the inherent radiosensitivity of the cells. The authors reported that glycogen accumulation was only observed for radiation resistant tumour cell lines compared to radiation sensitive tumour cell lines. Glycogen metabolism can be related to a host of signalling pathways associated with tumour progression. Furthermore, studies have suggested a link between glycogen accumulation and hypoxia^38^ as intracellular glycogen is thought to protect tumour cells against hypoxia and other forms of cellular stress.^39^

Raman spectroscopy has not been extensively used to assess radiation response in brain tumour cells. An early study by Lakshmi *et al.* showed the spectral changes of mice brain tissue following irradiation were similar to changes observed when the mice were subject to other external stressors, such as restraint and innoculation.^40^ Further work by Kumar *et al.* used Raman spectral signatures to predict the radiosensitivity of glioma stem-like cells.^26^ Their study used radiosensitising agents to alter the sensitivity of the cells to external beam radiation (XBR) treatment. Raman spectroscopy was then used to assess the radiation response of the cells between 6 to 48 hours following 8 Gy XBR treatment. This allowed Raman spectroscopy to be used as a method for predicting radiation resistance in the glioma cells.

Raman spectroscopy has been further used as a method to predict radiation resistance in tumour cells by evaluating radiation response.^26,28,37,41^ These studies used Raman spectroscopy to determine that radiation resistant and radiation sensitive cells have varying responses to X-ray radiation exposure. This highlights the potential for Raman spectroscopy to further investigate the factors which effect cellular radiation response such as the phase of the cell cycle during radiation exposure.

Cancer cells contain mutations which can affect growth signalling, allowing the cells to evade apoptosis.^42,43^ The control over proliferation exhibited in normal cells is lost in cancer cells, causing continuous proliferation and therefore continuous progression through the cell cycle. The aim of most cancer treatments is to control and halt the progression of malignant cells through the cell cycle in order to kill the cells.^44^ The position of a cell in the cell cycle is an important internal factor that effects the cellular radiosensitivity, as cellular radiosensitivity is dependent upon stage within the cell cycle (Fig. 1).^45^ In general, cells display radiation resistance towards the end of the G_1_ phase and into the S phase, whereas cells in the G_2_ phase and those going through mitosis, will display greater radiosensitivity. In addition, cells may possess greater radiation resistance at the very beginning of the G_1_ phase, but this is dependent on the overall length of this phase for specific cell types.^45^

**Fig. 1:**
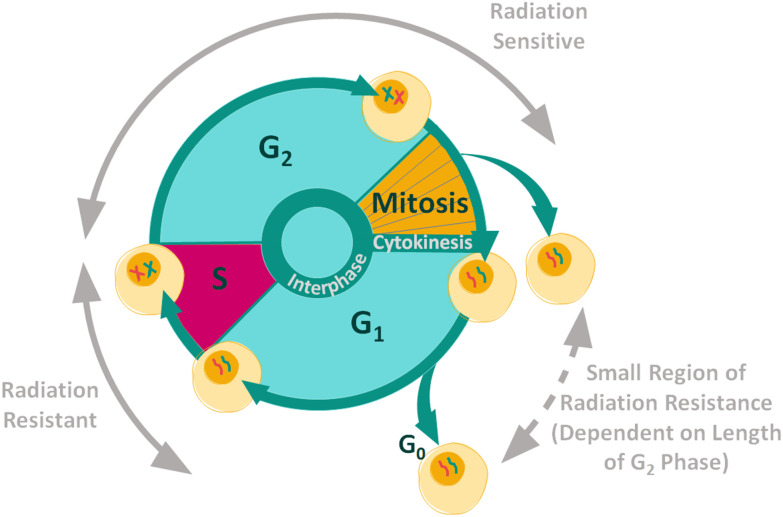
Radiation resistance and sensitivity changes through the cell cycle.^45–47^

The radiosensitivity of different phases of the cell cycle has been studied by irradiating synchronised populations of cells.^46–48^ However, comparison of the results is difficult due to the variation observed between cell types. Raman spectroscopy has been shown to be useful in the study of radiation response and radiation resistance in cancer cells. In addition, Raman spectroscopy has been used to discriminate between cells at different stages of the cell cycle.^49–53^ Therefore Raman spectroscopy shows promise as a method for the study of cell cycle induced radiation resistance. Here we use Raman spectroscopy to study radiation resistance in UVW human glioma cells to gain a better understanding of the effects of cell cycle synchronization on Raman spectral variation and thereby as a method for predicting radiation resistance.

## Materials & methods

### Cell culture

This study used the UVW (human glioma) cell line which is a cell line derived in the Beatson Institute for Cancer Research, UK. The cells were cultured in 75 cm^3^ tissue culture flasks in minimum essential medium (MEM) supplemented with 10% foetal bovine serum (FBS), 200 mmol L^−1^l-glutamine, 100 μg mL^−1^ penicillin–streptomycin and 2 μg mL^−1^ amphotericin B (Gibco, UK). The cells were incubated at 37 °C in a humidified 5% CO_2_ atmosphere. Prior to experiments, UVW cells were seeded at a concentration of 1 × 10^5^ cells into 6-well plates containing CaF_2_ coverslips for attachment and incubated at 37 °C in a humidified 5% CO_2_ atmosphere for 24 hours until exponential growth phase was reached at 70–80% confluency.

### Double thymidine block (DTB) cell synchronisation

A double thymidine block (DTB) treatment was used to synchronise the UVW cells at the G_1_/S boundary of the cell cycle.^54^ UVW cells were incubated with cell culture medium containing 2 mM thymidine for 18 hours. The treatment was then removed, the cells were washed with phosphate buffered saline (PBS) and incubated with thymidine-free cell medium for 9 hours. Cells were then treated again with 2 mM thymidine for 18 hours.

### X-ray treatment

Cells were irradiated at a 6 Gy dose in the 6-well plate while maintained in MEM. X-ray exposure was performed with an X-RAD 225 X-ray cell irradiation cabinet (Precision X-ray, USA) with a 225 kVp X-ray beam and a dose rate of 2.2 Gy min^−1^.

### Raman spectroscopy

UVW cells were fixed at 1 hour, 4 hours and 24 hours following radiation treatment with 4% paraformaldehyde (PFA) prior to Raman analysis. Individual Raman cells maps were collected using a Renishaw InVia confocal Raman microscope with a 532 nm Ng:YAG laser excitation source (maximum power of 50 mW at laser), 1800 l mm^−1^ grating, edge filter and a charge-coupled device (CCD) detector and a Leica 50× objective (N.A: 0.75). Renishaw WiRE 4 series software was used to collect sample spectra cell maps. All spectral maps were collected with 1 second per spot acquisition time, 21 mW laser power at sample (100%), 1 μm *X*/*Y* step size and spectral centre of 1300 cm^−1^. For each sample condition, maps were taken of at least 10 individual cells with at least 900 Raman measurements collected per cell. All experiments were carried out with three independent replicates and in total 43 cell maps were collected per sample group.

### Cell cycle analysis

UVW cells were detached from the flask into a single cell suspension and fixed with 70% EtOH. Cells were then incubated with 50 μg mL^−1^ ribonuclease A (RNase A) and 10 μg mL^−1^ propidium iodide (PI) to degrade the ribonucleic acid (RNA) and stain cellular DNA, respectively (full details in ESI†). Samples were incubated in a light-free environment at 4 °C for at least one hour prior to analysis. All flow cytometry experiments were performed using an Attune NxT flow cytometer with Attune NxT Software (Version 3.1.2). For cell cycle analysis, cells were analysed with 100 μl acquisition volume and a 100 μl min^−1^ flow rate with a minimum of 10 000 events analysed. Cell fluorescence was assessed using a blue 488 nm 50 mV laser and forward scatter (FCS), side scatter (SSC) and BL2 detectors (574/26 channel). Detector voltages were 140 V, 300 V and 320 V for FSC, SSC and BL2, respectively.

### γ-H2AX assay

UVW cells were detached from the flask into a single cell suspension and fixed with 4% PFA. UVW cells were permeabilised using Triton-X-100, resuspended in blocking buffer (0.1% Triton-X-100 and 0.5% bovine serum albumin (BSA) in PBS) and incubated with 100 μg mL^−1^ FITC-conjugated anti-phospho-histone H2AX (SER139) antibody (Millipore, UK) for 20 minutes at 4 °C, full details in the ESI.† Cells were then analysed by flow cytometry with a 90 μl acquisition volume and a 200 μl min^−1^ flow rate with a minimum of 10 000 events analysed. Cell fluorescence was assessed using a blue 488 nm 50 mV laser and forward scatter (FCS), side scatter (SSC) and BL1 detector (530/30 channel). Detector voltages were 90 V, 300 V and 265 V for FSC, SSC and BL1, respectively.

### Data processing

The Raman spectra collected were first pre-processed using WiRE 4.4 software to remove cosmic rays from the spectra using the built-in cosmic ray removal function. MATLAB software (R2017a) was used to further process the Raman spectra, using in-house MATLAB scripts. This software was used for noise reduction (nonlinear iterative partial least squares (NIPALS) decomposition), *x*-axis standardisation, baseline correction (rolling-circle filter (RCF) with radius 150 units), quality control and area under the curve (AUC) normalisation. The spectra were also truncated to 900–1770 cm^−1^ and the spectra were *x*-axis normalised to the phenylalanine peak at 1004 cm^−1^. The pre-processed spectra from each sample group were averaged and principal component analysis (PCA) was then performed using the in-built MATLAB PCA function. Random forest (RF) modelling was performed on PCA scores using the open source randomForest package in R software (Version 3.6.1). Variable importance was assessed using GINI index. GINI is a measure of how each variable contributes to the homogeneity of the nodes in the random forest. For example, each time a particular variable is used to split a node, the boundary value set on this variable will influence the resultant purity of the data split using this node. Variables which have a high value GINI are important factors in classifying the data correctly. ANOVA statistical analysis was carried out using GraphPad Prism 8 software (Version 8.4.2, GraphPad Software Inc., USA) with Bonferroni post-tests at a 95% confidence interval (CI) for the cell cycle data and with Wilcoxon rank sum test at 99% CI for PCA loadings. Full details of the data processing methods are described in the ESI.†

## Results & discussion

The position of cells in the cell cycle is known to influence the radiation response of cancer cells.^45–47^ Despite this, synchronising the cell cycle prior to the study of radiation response has not been extensively researched when assessing Raman spectroscopy as a method for monitoring cellular radiation response. Predicting the differences in radiation response resulting from different factors, including cell cycle variation, is an important consideration to patient tailored radiation therapy. In most types of cancer therapy, for example chemotherapy and radiation therapy, a typical treatment outcome is cell cycle arrest, leading to cell cycle synchronisation. As treatment is typically given over multiple fractions or doses it is important to understand how variation in cell cycle can affect treatment response. Raman spectroscopy has previously been shown to be useful as a method for monitoring cellular radiation response.^22–31^ Here we aimed to compare the radiation response of unsynchronised UVW cells and UVW cells synchronised to the G_1_/S boundary prior to radiation treatment using Raman spectroscopy. In doing so we aimed to determine whether Raman spectroscopy could be used as a tool for monitoring subtle cellular changes associated with cell cycle variation and radiation response within the first 24 hours following radiation treatment.

Cell cycle synchronisation can be used to harmonise a large population of cells at a particular stage of the cell cycle. Different methods of cell cycle synchronisation have been previously described including serum starvation,^55,56^ contact inhibition^52,57^ and mitotic selection.^52^ Cell synchronisation can also be achieved by the addition of agents such as thymidine,^52,54^ nocodazole^52^ and aphidicolin.^57^ For this study, four different synchronisation methods were investigated. These methods synchronised cells to the G_1_/S boundary by double thymidine block (DTB) treatment^54^ (Fig. S1B†), to the S phase by DTB treatment followed by fresh medium reincubation^52,54^ (Fig. S1C†), to the G_2_/M boundary by thymidine and nocodazole treatment^52,54^ (Fig. S1D†) and to the early G_1_ phase by thymidine and nocodazole treatment followed by fresh medium reincubation^52,54^ (Fig. S1E†). The full details of these synchronisation methods are shown in the ESI.† However, the most successful method was the DTB treatment. Thymidine reversibly incorporates into the DNA of cells preventing the cell from progressing from the G_1_ phase to the S phase of the cell cycle since it prevents DNA replication from occurring.^54^ The cells therefore become synchronised at the G_1_/S boundary of the cell cycle, which has been demonstrated to render the cells more radioresistant.^45^ Cell cycle analysis using fluorescence activated cell sorting (FACS) demonstrated that following treatment with the DTB, 71% of cells occupied the G_1_ phase of the cell cycle suggesting successful synchronisation (ESI – Fig. S1†). This was in comparison to a control, unsynchronised, cell population where approximately 50% of the cells were present in the G_1_ phase of the cell cycle.

Both unsynchronised and synchronised cells were then treated with 6 Gy XBR before samples were collected and fixed at 1 hour, 4 hours and 24 hours following radiation treatment for cell cycle analysis, Raman measurements and γ-H2AX analysis. First, cell cycle analysis was used to confirm the cell cycle distribution of the control and 6 Gy groups irradiated at each time point. The statistical significance of changes to cell cycle distribution, comparing the control to the irradiated cells, was obtained using two-way ANOVA analysis.

**Fig. 2:**
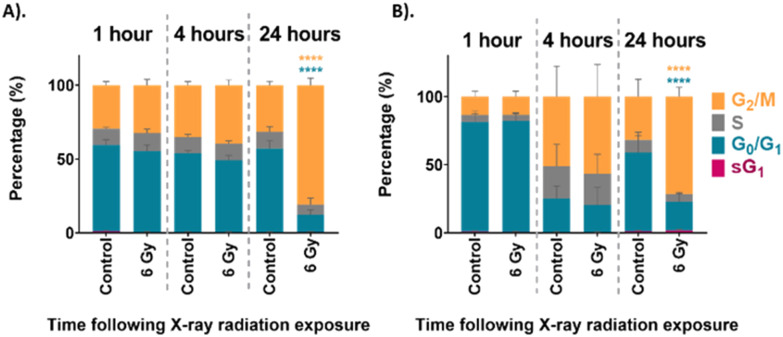
Cell cycle analysis of (A). unsynchronised UVW cells, and (B). synchronised UVW cells. FACS results comparing cell cycle distribution of control and 6 Gy irradiated cells at 1 hour, 4 hours and 24 hours following irradiation. The coloured bars represent percentage of cells in sG_1_ phase (pink), G_0_/G_1_ phase (blue), S phase (grey) and G_2_/M phase (yellow). Data presented as an average of three independent replicates (mean ± standard deviation). Two-way ANOVA compared the mean cell cycle phase population at each time point following irradiation to the untreated control cells. Statistical analysis was performed using two-way ANOVA with Bonferroni post-tests at 95% confidence interval (*p* > 0.05 = ns (not significant) and *p* < 0.0001 = ****).

The cell cycle distribution of the unsynchronised cells is shown in Fig. 2A. The unirradiated control sample at each time point showed a distribution of cells which would be considered normal for an asynchronous population with 59% in G_0_/G_1_ and 30% in G_2_/M phase. This distribution of the UVW cells in the different phases of the cell cycle did not change over the 24 hours assessed. On the other hand, the cells which were irradiated show a distinct change in cell cycle distribution over the 24 hours following irradiation. At the 1-hour time point the 6 Gy sample had a cell cycle distribution similar to that of the unirradiated control cells. After 4 hours following irradiation the 6 Gy sample showed a small, but not statistically significant, decrease in G_0_/G_1_ population and an increase in G_2_/M population compared to the control (*p* > 0.05). At the 24-hour time point there was a statistically significant increase in G_2_/M population between the control and irradiated samples (*p* < 0.0001), with 81% of cells occupying the G_2_/M phase of the cell cycle. This confirmed that the UVW cells exhibited cell cycle arrest in the G_2_/M phase 24 hours following radiation exposure.

The cell cycle distribution of the synchronised cells was then assessed using FACS analysis. These cells were first synchronised at the G_1_/S boundary by DTB treatment before exposure to 6 Gy XBR. The cell cycle progression of the synchronised cells is shown in Fig. 2B. At the 1-hour time point, approximately 80% of both the control and irradiated cells were in the G_0_/G_1_ phase of the cell cycle which confirmed that the synchronisation had been successful. At the 4-hour time point the cells showed that the distribution of cells had changed. Now the cells exhibited a lower population in the G_0_/G_1_ phase and a higher population in the G_2_/M phase demonstrating that cells had begun to move through the cell cycle. Finally, at the 24-hour time point the percentage of the control cell population in the G_0_/G_1_ phase had increased to 58% indicating that cells had continued to progress through the cell cycle. At this time point the cells displayed a distribution similar to the unsynchronised control cells observed in Fig. 2A. This could suggest that following G_1_/S boundary synchronisation by DTB treatment, the effects are short lived and cells become unsynchronised in the 24 hours following treatment. This is likely to be a result of the variation in the rate at which cells progress through the cell cycle when they are released from thymidine treatment. This is one factor that limits the use of cell cycle synchronisation as a method for the study of the different phases of the cell cycle. After 24 hours there was a statistically significant increase in the population of 6 Gy irradiated cells in the G_2_/M phase (72%) (*p* < 0.0001) which indicated that cells had arrested at the G_2_/M boundary because of radiation exposure.

Both the unsynchronised and synchronised UVW cells exhibited cell cycle arrest in the G_2_/M phase 24 hours following irradiation of the cells. The passage of cells to the next phase of the cell cycle is regulated at checkpoints throughout the cycle. This ensures that the DNA integrity is maintained preventing genetic flaws to be passed on to the next generation.^58^ Two checkpoints detect DNA damage in a cell, one at the G_1_/S transition and one at the G_2_/M transition.^59^ The checkpoint at the G_2_/M boundary prevents a cell entering mitosis if it is compromised. The extent of the G_2_/M arrest was significantly different between the unsynchronised and synchronised sample groups (*p* < 0.05). For the unsynchronised cells, 81% of the population occupied the G_2_/M phase 24 hours following radiation exposure compared to only 72% occupying the G_2_/M phase for the synchronised cells. This showed that in the synchronised cells fewer cells were experiencing arrest before mitosis and more cells were able to move through to the G_1_ phase. This suggested that the cells were more viable when irradiated following synchronisation at the G_1_/S boundary of the cell cycle which could suggest less DNA damage was induced. This confirmed that these cells display radioresistance in the G_1_/S phase of the cell cycle.^45^

**Fig. 3:**
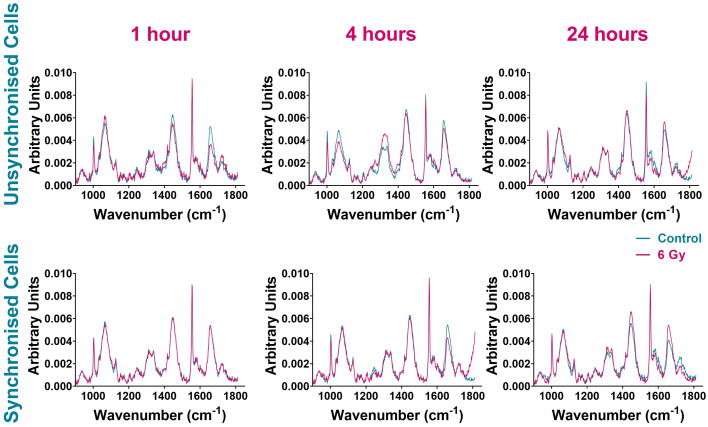
Average Raman spectra comparing control UVW cells (blue) and 6 Gy irradiated UVW cells (pink). Unsynchronised cells 1-hour, 4-hour and 24 hours following radiation exposure (left panel). Synchronised cells 1 hour, 4 hours and 24 hours following radiation exposure (right panel). Results are presented as an average spectrum of three independent replicates including 43 single cell maps in total per sample and each replicate contained at least 10 cell maps.

In combination with FACS analysis, Raman spectroscopy was assessed as a method for monitoring cellular changes associated with radiation response immediately following radiation exposure. Raman analysis was used to collect individual cell maps of both unsynchronised and synchronised UVW cells collected and fixed 1 hour, 4 hours and 24 hours following treatment (control and 6 Gy). Average Raman spectra were produced by averaging all spectra from three independent replicates totaling 43 cell maps per treatment group; each map contained at least 900 individual cell spectra. The average Raman spectra of each sample group are shown in Fig. 3 and Raman peak assignments are shown in Table 1.

**Table 1:** Wavenumber assignments for main peaks in Raman spectra^60^

Wavenumber	Assignment
963 cm^−1^	Protein, tyrosine^61^
1004 cm^−1^	Phenylalanine^23^
1032 cm^−1^	C–H in-plane bending mode of phenylalanine^22,52^
1056 cm^−1^	Lipid^62^
1065 cm^−1^	Protein C–N stretch, skeletal C–C stretch of lipids^63^
1235 cm^−1^	Amide III^64,65^
1270 cm^−1^	Phospholipids, amide II (proteins), C–N alpha helix proteins, C=C fatty acids^60,66^
1320 cm^−1^	Guanine, amide III^22,34^
1330 cm^−1^	DNA, phospholipids^67^
1340 cm^−1^	Amide III & CH_2_ wagging vibrations (glycine backbone, proline side chain), C–H deformation (protein)^22,52,68^
1427 cm^−1^	CH_2_ and CH_3_ bending of methyl bonds in nucleic acids
1445 cm^−1^	Collagen, phospholipids, CH_2_ bending modes (protein & lipids), CH_2_ deformation^22,65^
1555 cm^−1^	Amide II, tryptophan^60^
1596 cm^−1^	Amide I of protein (C=O stretching)^69^
1614 cm^−1^	Tyrosine, tryptophan^22,52^
1631 cm^−1^	Amide III^64^
1656 cm^−1^	Amide I *ν*(C=O), C=C lipids, tryptophan^22,52^

The spectra of unsynchronised cells show variation in Raman intensity between the control and irradiated samples at all time points (Fig. 3). The greatest variation was observed at 1-hour and 4 hours post irradiation. Differences in signal intensity were observed between the control and 6 Gy cells for Raman peaks at 1004 cm^−1^ and 1032 cm^−1^ assigned to phenylalanine, 1320 cm^−1^ and 1340 cm^−1^ assigned to amide III and 1555 cm^−1^ and 1656 cm^−1^ assigned to tryptophan and amide I (Table 1). The least variation was seen in the 24-hour sample (Fig. 3, right upper panel), where the control and 6 Gy samples spectra were the most similar although small differences in signal intensity between the control and 6 Gy samples were observed at 1555 cm^−1^ and 1656 cm^−1^, associated with tryptophan and amide I.

The spectral variation observed between the control and 6 Gy samples was different for the synchronised cells (Fig. 3). These cells were synchronised to the G_1_/S boundary prior to radiation treatment; thus the majority of the cells were considered to be in the same phase of the cell cycle. One hour post irradiation there was very little difference between the control and 6 Gy sample spectra, suggesting that there was little response in cells immediately after irradiation. After 4 hours following irradiation however, the spectra of the control and 6 Gy samples displayed more spectral variation. This variation was observed with differences in peak intensities between the samples at 1004 cm^−1^ and 1656 cm^−1^ indicating different expression of phenylalanine and amide I signals. Variation between the control and irradiated sample spectra were still present in the 24-hour sample (Fig. 3). The sample spectra showed differences in peak intensities between the control and 6 Gy samples at 1445 cm^−1^ and 1656 cm^−1^. This demonstrated variation in collagen, phospholipids, tryptophan and amide I at this time point.^37,70^

Overall, the average cell spectra comparing the control and 6 Gy irradiated cells suggested that there was a difference between the sample spectra at each time point for both unsynchronised and synchronised cells. However, it was unclear if the variation observed was solely caused by radiation response in the cells or if there were contributions from differences in cell cycle distribution. Principal component analysis (PCA) combined with random forest (RF) classification and variable selection, was used to evaluate the variation associated with radiation response. The sample spectra (Fig. 3) showed a large feature at 1555 cm^−1^ which was assigned to instrumental background. A Raman peak at 1555 cm^−1^ in cell spectra could be assigned to tryptophan/amide I (Table 1). A study by Van Nest *et al.* reported changes to the tryptophan signal (1555 cm^−1^) in the Raman spectra of non-small cell lung cancer (NSCLC) tissue xenografts as a result of radiation response to 15 Gy XBR.^36^ Furthermore, tryptophan metabolism has also been associated with radiation-induced immunosuppression involving immune checkpoint reactivation.^70^ However, the appearance of the peak at 1555 cm^−1^ was very sharp (Fig. 3) and this feature was dominant in the Raman spectrum. These characteristics would be unusual in a cell spectrum, therefore this feature was assigned to instrumental background as off-sample spectra were shown to contain the same peak (ESI – Fig. S2†). For this reason, this peak was not used for evaluation of radiation response and the PCA included this background spectrum to minimise its contribution to the variation found between sample groups.

Prior to PCA, all the spectra in each cell map were averaged to give one spectrum per cell analysed. An average cell spectrum was used as a large variation would be observed between cell spectra from the same cell depending on their location inside the cell. Using an average cell spectrum removes this variability and allows comparison between cells under different treatment conditions.^71,72^ PCA was first performed using all the data from all time points in order to compare the control and 6 Gy irradiated cells in each sample condition. The first ten PC loadings generated for this PCA are shown in the ESI (Fig. S3A†).

Following PCA, RF analysis was used to determine which PC loadings described the variation between irradiated and non-irradiated samples for each time point. An advantage of using RF for classification is the ability to evaluate the importance of the identified predictor variables for correct classification.^73,74^ The ten most important PCs to correctly classify the data as irradiated and non-irradiated for 1, 4 and 24 hours post irradiation are shown in Fig. S3.† Using RF to classify the data into the different treatment groups identified PC3 as the most important loading, followed by PC14, PC8 and then PC7. Full results of the RF analysis are shown in the ESI – Fig. S2.† Out-of-bag (OOB) error was calculated to determine the prediction error of the RF analysis. In this analysis, the average out-of-bag error was 58 ± 4%, 49 ± 2% and 29 ± 2% for 1-hour, 4-hour and 24-hour time points, respectively. This showed that the model could more accurately assign the samples into the correct groups as the time from treatment increased. The RF model could not accurately classify the samples at both the 1-hour or 4-hour time point. However, it was able to assign the samples into groups for the 24-hour time point samples with over 70% accuracy. This could suggest that the Raman signatures associated with radiation response are too subtle in the early hours following treatment.

The most important variable for classification was PC3 and this loading described 12.6% of the overall variance within the data set, while PC14, PC8 and PC7 accounted for 0.3%, 0.9% and 1.3% respectively (Fig. S3†). Although the percentage variance of these loadings are lower compared to earlier loadings such as PC1 and PC2, RF identified this PC loading as the most important for describing the difference between the untreated and treated samples. Despite earlier PC loadings describing larger percentage variance, in this case it is likely that this variance results from heterogeneity within the samples due to other factors unrelated to treatment response. For example, PC1 was shown to have features at 1032, 1445 and 1656 cm^−1^ (Fig. S2A†) indicating variation in phenylalanine, CH_2_ bending modes of proteins and lipids, amide I, C=C of lipids and tryptophan (Table 1). The PC2 loading similarly showed features at 1032 and 1656 cm^−1^ likely accounting for similar variations. This variation could be a result of differences in cell cycle position between cells of the same samples since the RF analysis did not find that these PC loading describe variation between sample groups. Additionally, these PC loadings may describe the difference in radiation response between each time point, for example the degree of radiation response between the 1-hour and 4-hour time point, as this may be the largest variation when considering the dataset as a whole. A previous Raman study by Tollefson *et al.* demonstrated that by using PC8 and PC10, which were relatively low in the list of overall variance, they were able to differentiate prostate cancer patients who would go on to develop distant metastasis *versus* those who did not.^75^ Although lower PC loadings are not often considered for further analysis, this highlights the importance of assessing PC loadings with smaller contributions to the overall variance between sample groups. The aim of using supervised learning techniques such as RF in combination with unsupervised methods, such PCA, is to extract information from the spectra which otherwise would be obscured by the inherent heterogeneous nature of the data.^76^

**Fig. 4:**
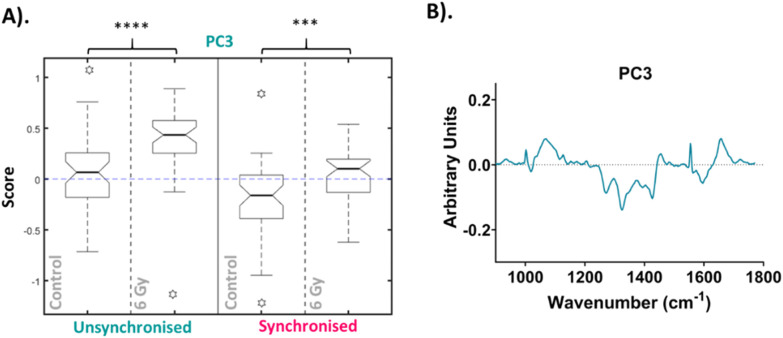
Principal component analysis (PCA) of average Raman spectra for 24-hour time point. (A). Box plot of PC3 median loading scores at the 24-hour time point. Box plot compares control cells and 6 Gy irradiated cells for unsynchronised UVW cells (blue) and synchronised UVW cells (pink). Centre point of box represents median value, notches represent the 25th and 75th percentile, whiskers represent the 5th and 95th percentile and stars represent outliers. (B). PC3 loading from PCA comparing control and 6 Gy samples for all time points following 6 Gy XBR exposure for unsynchronised and synchronised UVW cells. Statistical analysis was performed using a two-way ANOVA with Wilcoxon rank sum test at 99% confidence interval (*p* > 0.05 = ns (not significant), *p* < 0.001 = *** and *p* < 0.0001 = ****).

The median scores on the PC3 loading were plotted as a Tukey style box plot for the unsynchronised and synchronised samples at each time point, 1 and 4-hour time points are shown in the ESI as classification accuracy was poor (Fig. S4†). The Tukey box plots for the PC3 scores at the 24-hour time point are shown in Fig. 4 and the box plots for PC14, PC8 and PC7 scores are shown in the ESI (Fig. S5†). Using a Tukey box plot to visualise the data allowed the differences in scores from one PC loading to be assessed and allowed comparison of the scores attributed to a given PC loading between the sample groups. For each PC loading, the median score was compared for the control and 6 Gy irradiated cells for both unsynchronised and synchronised sample groups. At the 24-hour time point, the median scores on the PC3 loading were significantly different when comparing the control and irradiated cells for both the unsynchonised and synchronised cells (*p* < 0.001). For both sample groups the median PC3 score was higher for the irradiated cells compared to the control cells. The PC3 loading was then used to assign the changes in Raman spectra for the different sample groups. The PC3 loading plot is shown in Fig. 4. The PC3 loading had large features at 1004, 1065, 1270, 1320, 1427, 1596 and 1656 cm^−1^. The feature at 1004 cm^−1^ was assigned to phenylalanine and the feature at 1065 cm^−1^ was assigned to the C–C stretch of lipids. The features at 1270, 1320 and 1596 & 1656 cm^−1^ were assigned to amide II, amide I and amide III features, respectively (Table 1). Finally, the band at 1427 cm^−1^ was assigned to CH_2_ and CH_3_ bending of methyl bonds in nucleic acids (Table 1). This feature could be a result of changes to DNA within the cells, as DNA damage is the main outcome of radiation treatment. Overall, the features contributing to the PC3 loading were predominantly amides from proteins with some contribution from lipids. Therefore, Raman analysis found that changes in lipids, but predominantly proteins, accounted for the radiation response in UVW cells. UVW cells have previously not been studied by Raman spectroscopy, however changes to lipid expression following radiation response has been shown by Paidi *et al.* who demonstrated changes to lipid and collagen content in both lung (A549 cell line) and head and neck (UM-SCC-47 and UM-SCC-22B cell lines) cancer cells xenografts following 8 Gy radiation.^37^

To improve the classification accuracy of the RF analysis, PCA was then used to assess the variation between samples types at each time point separately. From the PCA of the 1-hour time point data, the first ten PC loadings are shown in the ESI (Fig. S6†). RF was then used to determine the most important variables for each time point. For the 1-hour time point, the OOB error for classification was 46 ± 2%, although reduced compared to the previous analysis, a large error was still observed. The ten most important PC loadings for classification are shown in the ESI (Fig. S6†). The RF model found that for the 1-hour time point the PC4 loading was the most important variable followed by the PC1 loading, full details are described in the ESI (Fig. S6†). Since the error in classification was high for the 1-hour time point, this time point was not assessed for radiation response (Fig. S7†). This suggested that 1 hour is too early for Raman spectroscopy to be used to detect the subtle changes associated with radiation response or that the method for assessing the Raman data is insufficient for identifying these subtle changes. This is in line with current literature, wherein most studies do not evaluate radiation response earlier than 24 hours. Further studies would be required in order to assess radiation response as early as 1 hour following radiation exposure.

**Fig. 5:**
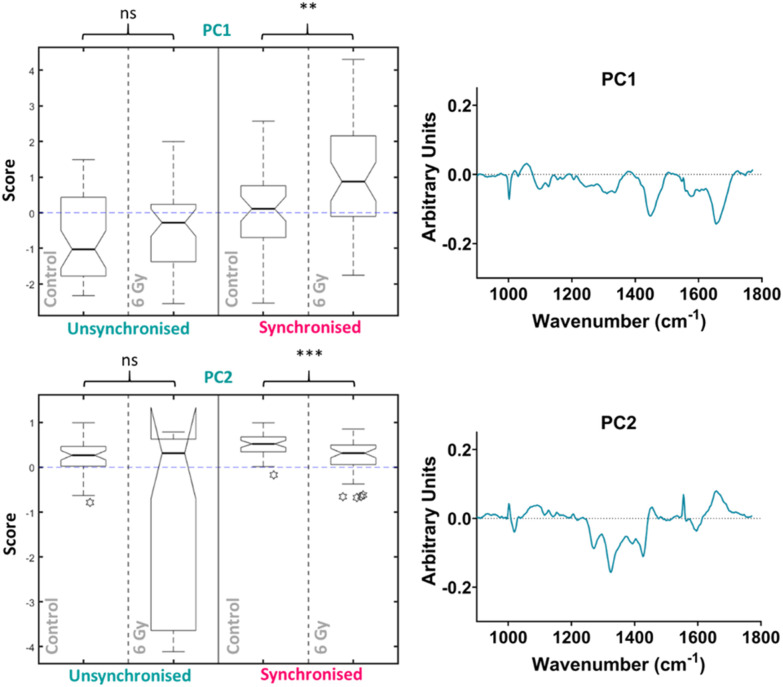
Principal component analysis (PCA) of average Raman spectra for just the 4-hour time point showing the box plots and PC loadings for PC5 and PC1. Box plot compares control cells and 6 Gy irradiated cells for unsynchronised UVW cells (blue) and synchronised UVW cells (pink). Centre point of box represents median value, notches represent the 25th and 75th percentile, whiskers represent the 5th and 95th percentile and stars represent outliers. PC5 and PC1 loading from individual time point PCA comparing control and 6 Gy samples for the 24-hour time point following 6 Gy XBR exposure for unsynchronised and synchronised UVW cells. Statistical analysis was performed using a two-way ANOVA with Wilcoxon rank sum test at 99% confidence interval (*p* > 0.05 = ns (not significant), *p* < 0.01 = ** and *p* < 0.001 = ***).

PCA was then performed on the 4-hour time point data alone, the first ten PC loadings are shown in the ESI (Fig. S6†). The RF model, which was used to classify the data as control *vs.* 6 Gy irradiated for each group (unsynchronised and synchronised), showed that for the 4-hour time point the OOB error was 40 ± 3% therefore showing better classification compared to the PCA analysis which considered all the data together. This showed that the model could classify the data accurately 60% of the time. The ten most important PC loadings for classification are shown in the ESI (Fig. S6†). The RF found the PC1 loading to be the most important variable, followed by the PC2 loading, full details are described in the ESI (Fig. S6†). The box plot of the PC1 scores showed that there was an increase in PC1 median score for the irradiated cells compared to the control for the unsynchronised sample group, however this difference was not significant (*p* > 0.05) (Fig. 5). The same trend was observed for the synchronised cells which showed a significant difference between the control and 6 Gy irradiated samples in the synchronised samples groups (*p* < 0.01). Although the variation in the median PC1 score observed for the unsynchronised cells was not significant, the larger OOB error observed for classification at the 4-hour time point could explain why the variation observed was not entirely related to radiation response. The PC1 loading showed main features at 1004, 1056, 1445 and 1656 cm^−1^ which could be assigned to phenylalanine, lipid, CH_2_ bending modes of lipid and protein and amide I/lipids, respectively. The box plot for the PC2 scores for the 4-hour time point also showed that there was no significant difference between the control and 6 Gy irradiated samples in the unsynchronised sample group (*p* > 0.05). However the PC1 loading did describe a significant difference between the control and 6 Gy irradiated samples in the unsynchronised sample groups (*p* < 0.01). The PC2 loading was almost identical to the PC3 loading from the PCA described for the data all together (Fig. 4) and were shown to have high positive correlation (*r* = 0.95) using Pearson's linear correlation coefficient. This showed features at 1004, 1065, 1270, 1320, 1427, 1596 and 1656 cm^−1^ (Table 1). Overall, the features were predominantly from amides associated with proteins and some contribution from lipids. These results showed that at the 4-hour time point, PCA was able to determine a significant difference in the radiation response of the synchronised but not in the unsynchronised cells. This could suggest that by removing the variation in sample spectra caused by cell cycle position, we are able to evaluate the radiation response as early as 4 hours following XBR treatment.

The significant difference between the control and irradiated samples in the synchronised cells could be explained by the positioning in the cell cycle at the point of radiation exposure. The response to radiation exposure varies depending on the phase of the cell cycle in which the cell occupies.^46–48^ As the cells are synchronised in the same phase, this removes variation in response relating to position in the cell cycle. However, in the unsynchronised cells the degree of radiation response will differ depending on the phase in which they were irradiated. Thus, at an early time point, like 4 hours after radiation, the variation in the unsynchronised cells may lead to less successful classification.

UVW cells have a doubling time of approximately 29 hours.^77^ The longest phase of the cell cycle is the G_1_ phase and may last up to 12 hours, the S phase will last between 5–8 hours and the G_2_ phase will be shorter at 4–5 hours in length.^78^ Finally, mitosis (M) lasts only around 1–2 hours. Cell cycle analysis showed that at the 4-hour time point, a large proportion of the synchronised cell population (>50%) were in the G_2_/M phase of the cell cycle (Fig. 2B). This confirmed that at this time point the cells had moved through the S phase. The mechanisms of DNA damage and repair will ultimately lead to cell cycle arrest at the G_2_/M boundary of the cell cycle.^79^ We observed in both the unsynchronised and synchronised sample groups that by 24 hours post radiation a large population of cells had reached cell cycle arrest at G_2_/M (Fig. 2). When cells were synchronised to the G_1_/S boundary prior to irradiation, the majority will reach the G_2_/M phase quicker and therefore experience an earlier cell cycle arrest. This could result in a more prominent difference in radiation response at the 4-hour time point compared to the unsynchronised cells. For the unsynchronsied cells, at the 4-hour time point a large proportion of the cells (>50%) remain in the G_1_ phase of the cell cycle (Fig. 2A). At this stage, these cells may not have reached the checkpoint prior to the S phase, therefore radiation response, such as DNA repair, may not have been instigated. The degree of radiation response in these cells is likely to differ to cells at a later stage of the cell cycle. This could explain why a larger radiation response is observed in the synchronised cells 4 hours post irradiation and by synchronising the cells to the G_1_/S boundary we are inducing a quicker radiation response.

**Fig. 6:**
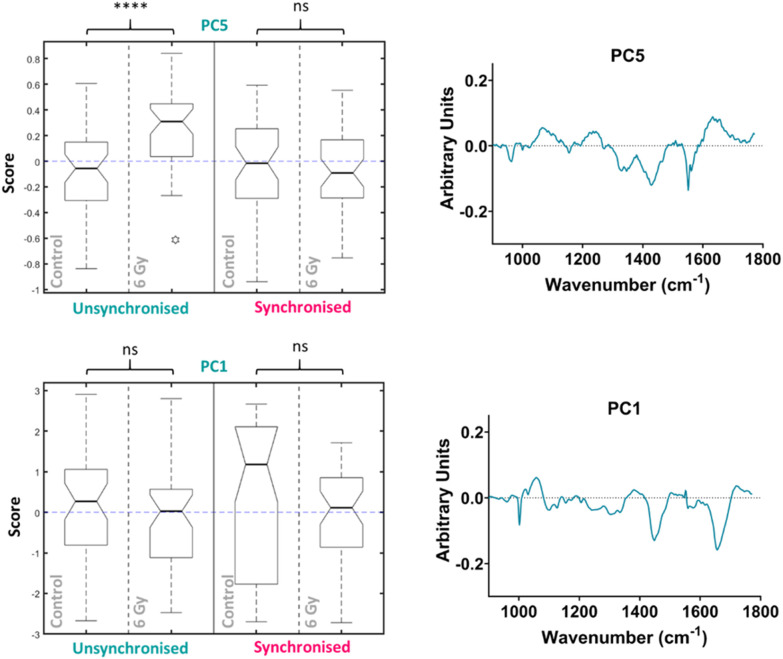
Principal component analysis (PCA) of average Raman spectra for just the 24-hour time point showing the box plots and PC loadings for PC5 and PC1. Box plot compares control cells and 6 Gy irradiated cells for unsynchronised UVW cells (blue) and synchronised UVW cells (pink). Centre point of box represents median value, notches represent the 25th and 75th percentile, whiskers represent the 5th and 95th percentile and stars represent outliers. PC5 and PC1 loading from individual time point PCA comparing control and 6 Gy samples for the 24-hour time point following 6 Gy XBR exposure for unsynchronised and synchronised UVW cells. Statistical analysis was performed using a two-way ANOVA with Wilcoxon rank sum test at 99% confidence interval (*p* > 0.05 = ns (not significant) band *p* < 0.0001 = ****).

PCA was then performed on the 24-hour time point data alone, the first ten PC loadings are shown in the ESI (Fig. S6†). Using the RF model, the 24-hour time point once again showed the best OOB error following classification of control *versus* 6 Gy irradiated cells for both unsynchronised and synchronised groups with an error of 35 ± 4%. This showed that the model was able to classify the sample correctly into treatment groups 65% of the time. The ten most important PC loadings found by the RF model for classification are shown in the ESI (Fig. S6†). The RF model found PC5 loading as the most important variable for classifying the samples into each group, followed by PC1. A Tukey box plot was used to show the difference between the sample groups for the PC5 loading, the box plot is shown in Fig. 6. The PC5 loading box plot showed a significant difference between the control and irradiated cells in the unsynchronised cells (*p* < 0.0001) but no significant difference between the control and irradiated cells un the synchronised sample group (*p* > 0.05). The PC5 loading had Raman features assigned to protein (963 cm^−1^ & 1429 cm^−1^), amide III (1235 cm^−1^ & 1631 cm^−1^), DNA and phospholipids (1330 cm^−1^) (Table 1). A Tukey box plot was used to show the difference between the sample groups for the PC1 loading, the box plot is shown in Fig. 6. The PC1 box plot did not show a significant difference between the control and irradiated cells for either the unsynchronised or synchronised samples (*p* > 0.05), therefore this PC loading was not used for evaluation of radiation response (Fig. 6).

Overall, this analysis showed that at 24 hours following irradiation PCA and RF could be used to show a difference in radiation response in the unsynchronised cells and this difference was a result of mostly changes to protein expression in the cells. The individual PCA analysis of the data from the 24-hour time point did not find significant difference between the control and treated in the synchronised cells. This was the opposite of what was found at the 4-hour time point. By synchronising the cells at the G_1_/S boundary prior to irradiation it would be expected that radiation resistance could be induced. At the 24-hour time point the most significant difference was observed in the unsynchronised cells. These cells should be more radiation sensitive which explains the larger radiation response observed. These cells were also shown to have greater cell cycle arrest at the G_2_/M boundary compared to the synchronised cells at this timepoint (Fig. 2). Furthermore, the model was also able to classify the cells into sample groups with more accuracy at the 24-hour time point compared to the earlier time points which suggested the changes in the cells related to radiation response are most prominent at 24 hours post irradiation. This is likely why the PCA, which considered all time points together, was able to classify the 24-hour samples with a higher accuracy than the earlier time points since at this time point the radiation response is more pronounced (Fig. 4).

Raman analysis was paired with the evaluation of cellular DNA damage and repair using γ-H2AX analysis to further elucidate radiation response within UVW cells. Exposure of the UVW cells to a 6 Gy dose of XBR would cause breaks in the DNA of the cells. The DNA damage and repair of cells was analysed through detection of SER139 phosphorylated γ-H2AX, a histone phosphorylated in response to DNA damage, by flow cytometry.^80,81^ The measured fluorescent signal corresponded to the level of γ-H2AX in the cells which directly relates to the amount of DNA damage in the cells. The γ-H2AX levels in irradiated cells was compared to the untreated control cells giving the fold changes of signal and therefore the degree of DNA damage in the irradiated UVW cells.

**Fig. 7:**
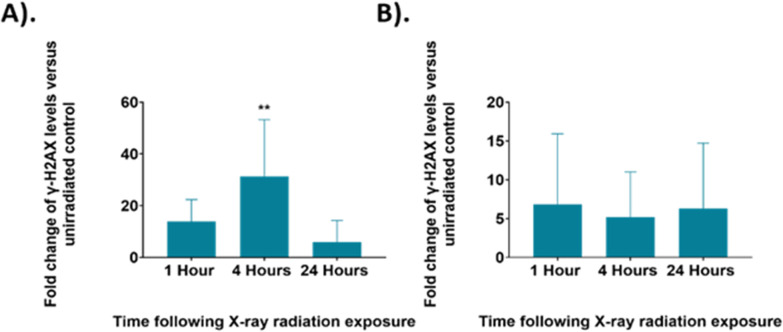
The fold change in γ-H2AX levels in irradiated UVW cells compared to an untreated control at each time point. (A). Unsynchronised UVW cells. (B). Synchronised UVW cells. Results are presented as an average of three independent experiments (mean ± standard deviation). One-way ANOVA compared the mean g-H2AX level fold change at each time point following irradiation to the untreated control cells. Statistical analysis was performed using one-way ANOVA with Bonferroni post-tests at 95% confidence interval (*p* > 0.05 = ns (not significant) and *p* < 0.01 = **).

The γ-H2AX levels were assessed at the same time points following irradiation as the Raman analysis for control and 6 Gy irradiated cells in both unsynchronised and synchronised sample groups (Fig. 7). The unsynchronised cells had a ∼14-fold increase in γ-H2AX levels at 1-hour following irradiation (*p* > 0.05) and a significant 31-fold increase in γ-H2AX levels 4 hours following irradiation compared to the non-irradiated control cells (*p* < 0.005). At 24 hours following irradiation, there were lower γ-H2AX levels, ∼6-fold change in γ-H2AX levels, in the irradiated samples compared to the non-irradiated control (*p* > 0.05). This showed that DNA damage increased within the first 4 hours following irradiation and then by the 24-hour time point DNA damage had been resolved. This decrease in DNA damage could be a result of DNA repair mechanisms being activated in the first 24 hours following irradiation or that cells which had sustained unrepairable damage were removed from the sample population due to apoptosis. The standard deviation observed from these results was high, which suggested that there was large variation between the DNA damage observed in each replicate. This method of assessing DNA damage using flow cytometry is less commonly used and γ-H2AX levels are usually quantified by fluorescence microscopy techniques by counting H2AX foci within a cell to quantify DNA double strand breaks.^81,82^ Therefore, further optimisation may be required to improve sensitivity of this method.

The synchronised cells had a different DNA damage and repair profile over the 24 hours following irradiation. At the 1-hour time point there was a ∼7-fold change in γ-H2AX levels, this decreased to a 5-fold change at the 4-hour time point and to a 6-fold change at the 24-hour time point, although the changes in γ-H2AX levels were not significant compared to the control (*p* > 0.05). Overall, these results showed similar DNA damage across all time points. Additionally, the γ-H2AX levels observed for the synchronised cells are overall lower than those observed in the unsynchronised cells, suggesting that less DNA damage occurred in the synchronised cells and therefore displayed greater radiation resistance. The standard deviation of these samples was also high suggesting there was a large variation between the DNA damage observed for each replicate. As with the FACS analysis, shown in Fig. 2, these results suggested that the G_1_/S boundary synchronised cells displayed radioresistance.^45^ By synchronising the cells to the G_1_/S boundary we therefore induced radiation resistance, and this resulted in the lower DNA damage observed in the synchronised cells. In addition, synchronising cells before the S phase, and therefore prior to DNA replication, would result in the average DNA content across the cells being lower compared to an unsynchronised population. A lower DNA content across the population would also explain why less DNA damage was observed.

The γ-H2AX results were compared to the results from the Raman analysis (Fig. 3–6), but the Raman analysis did not show a similar trend across the three time points. The Raman analysis showed changes primarily in cellular proteins and lipid whereas changes to DNA signal were not observed. Raman spectra from cells is dominated by protein and lipid signals, therefore, it may be expected that subtle changes to DNA, such as damage induced by radiation exposure, would not be observed. The γ-H2AX analysis for the unsynchronised cells showed largest DNA damage at 4 hours post irradiation (Fig. 7A). This then subsequently decreased by the 24-hour time point, suggesting DNA repair took place between 4 hours and 24 hours post radiation. The mechanism of DNA damage repair requires a large number of proteins responsible for double strand break detection, chromatin remodeling and DNA repair therefore it would be expected that the expression of proteins across the cell would change as the DNA is being repaired.^83^ The radiation response observed by Raman spectroscopy with PCA was significant at 24 hours in PC5 of the unsynchronised cells (Fig. 6). The main features present in the PC5 loading for the 24-hour time point were protein, amide III, DNA and phospholipids. Thus, the changes observed at this time point could account for the proteins required to facilitate the DNA repair between the 4-hour and 24-hour time point. A key feature that was shown to contribute to radiation response in some previous studies was glycogen.^24,30,31,34,36^ However, in this work no glycogen features were observed. Most previous studies observed glycogen accumulation 24 hours or longer after irradiation, although following a dose of 15 Gy Van Nest *et al.* did report glycogen accumulation as early as 2-hours following irradiation, although the dose used was much higher than that used in this study.^36^ Additionally, the most commonly assigned glycogen peak is around 473 cm^−1^ which was outside of the Raman range analysed in this study. The results presented could suggest that the first 24 hours after radiation exposure may be too soon to observe glycogen accumulation for UVW cells. Glycogen accumulation is also linked to radiation resistance in cells and therefore may not be present in cells which display radiosensitivity.^24^ The cell survival fraction (SF) describes the fraction of cells which maintain reproductive integrity following radiation exposure at a particular dose (*e.g.* 2 Gy – SF_2 Gy_). Although not extensively studied, previous work considered UVW cells to be moderately radioresistant (SF_2 Gy_ = 0.56),^84^ therefore the lack of glycogen accumulation in these cells contradicts previous studies which link glycogen accumulation to a cell's radiation resistance.^24,30,31,34,36^ The previous study by Van Nest *et al.* (2018) also did not find glycogen accumulation following radiation treatment of human breast adenocarcinoma xenografts. Their study, in agreement with this study, found spectral changes related to proteins including, phenylalanine and amide bands following irradiation, however these were not linked to specific biological processes.^35^ This study of radiation response in UVW cells presents similar findings with changes to protein features in the Raman spectra. Using γ-H2AX analysis in combination with Raman analysis was not conclusive in allowing similarities to be drawn between the spectral response and the level of DNA damage. However, the results suggested that the cellular protein changes observed at the 24-hour time point could be associated with the DNA damage pathways and repair mechanisms. Additionally, this work demonstrated that radiation resistance could be induced by controlling the cell cycle prior to radiation exposure which is a key consideration of cancer therapy and that by using Raman spectroscopy the differences in radiation response could be assessed.

## Conclusion

In this study Raman spectroscopy was used to assess radiation response in UVW human glioma cells cultured *in vitro*. The sensitivity of Raman spectroscopy for detection of subtle biochemical changes within the cells allowed evaluation of the early stages of radiation response as early as four hours following 6 Gy XBR treatment. The Raman spectral changes observed following radiation exposure were assigned to specific amino acids associated with proteins, including phenylalanine, and protein signals from amides. Using Raman spectroscopy in combination with γ-H2AX analysis suggested the protein changes observed in the synchonised cells were unlikely directly associated with the DNA damage and repair mechanisms of the cells. However, the protein changes observed, particularly between 4 hours and 24 hours post radiation treatment in unsynchronised cells could be associated with DNA repair pathways. Although Raman spectroscopy has been used previously to assess radiation response, few studies focus on the initial effects of this treatment compared to the longer-term radiation induced changes in cells. In the clinic, radiation treatment is often given daily in fractionated doses therefore monitoring the effects even after a short time, for example the first 24 hours following treatment, is equally important in assessing the long-term effects. In addition, cancer treatment often causes cell cycle arrest and therefore induces cell cycle synchronisation. Changes to the cell cycle during the course of a treatment regime will therefore influence the patients’ response, highlighting cell cycle position as an important factor to consider and monitor when studying radiation response. Raman spectroscopy was used to demonstrate the difference in radiation response between cells occupying different phases of the cell cycle at the time of irradiation. This work was able to show different levels of radiation resistance between cells and confirm that cell cycle variation influences the response of UVW cells to radiation exposure. To the best of our knowledge, this is the first report using Raman spectroscopy to monitor radiation response in synchronised cells and the results suggested that cell synchronisation is an important factor to take into account in future studies. By evaluating factors that induce radiation resistance in cells, a better understanding can be gained about radiation treatment. In addition, Raman spectroscopy has been shown to be a useful tool in differentiating different degrees of treatment response, which is important when investigating patient tailored radiation therapy. This highlights a need to further investigate the radiation response of cells at different phases of the cell cycle and other factors which induce radiation resistance.

## Author contributions

The manuscript was written through contributions of all authors. All authors have given approval to the final version of the manuscript.

## Conflicts of interest

The authors declare no competing financial interest. The research data associated with this paper is available at the following link: https://doi.org/10.15129/9ba64ab3-ce67-45d2-84ae-d2e94209713c.

## Supplementary Material

AN-148-D3AN00121K-s001

AN-148-D3AN00121K-s002

AN-148-D3AN00121K-s003

AN-148-D3AN00121K-s004

AN-148-D3AN00121K-s005

AN-148-D3AN00121K-s006

AN-148-D3AN00121K-s007

AN-148-D3AN00121K-s008

AN-148-D3AN00121K-s009
