# Supplementary figures and images for: Understanding radiation response and cell cycle variation in brain tumour cells using Raman spectroscopy
Source: Analyst. 2023 May 1;148(11):2594–608. doi: 10.1039/d3an00121k (PMC10228487; doi:10.1039/d3an00121k)

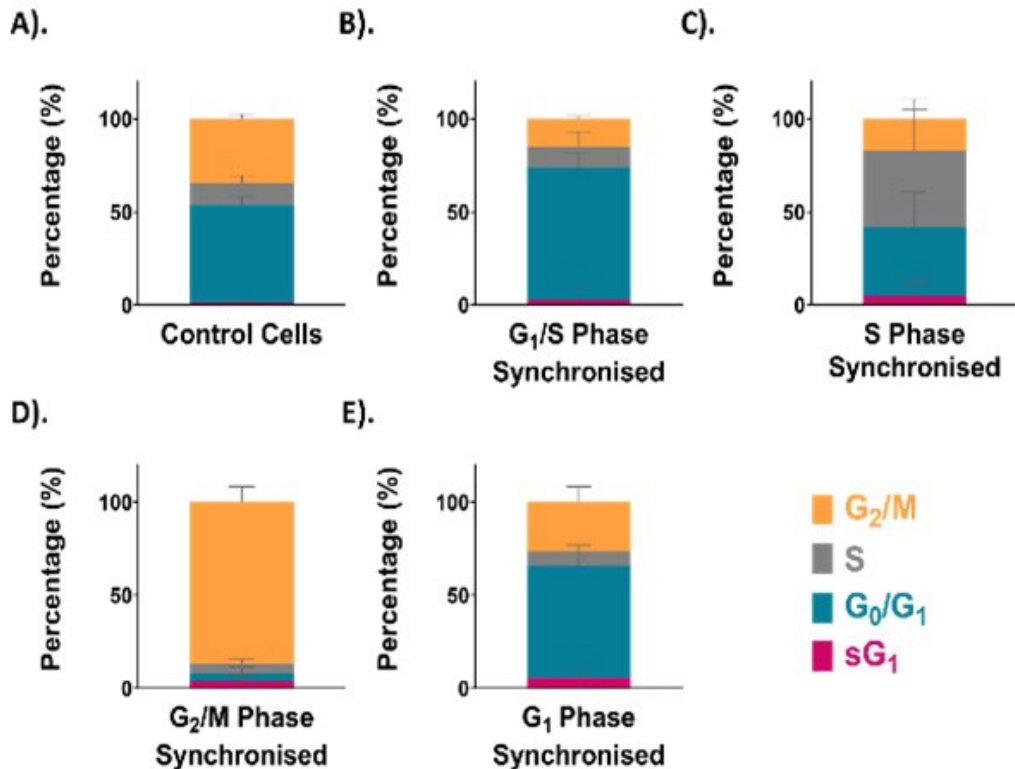

Supplement: AN-148-D3AN00121K-s002 [file AN-148-D3AN00121K-s002.pdf]

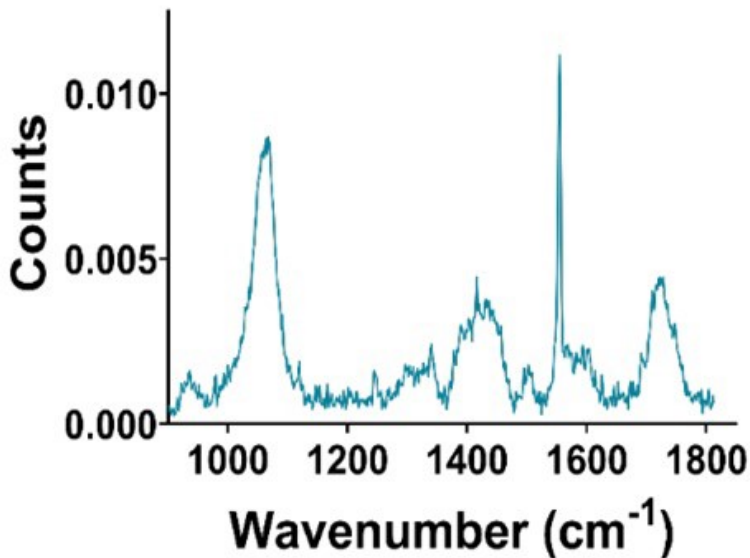

Supplement: AN-148-D3AN00121K-s003 [file AN-148-D3AN00121K-s003.pdf]

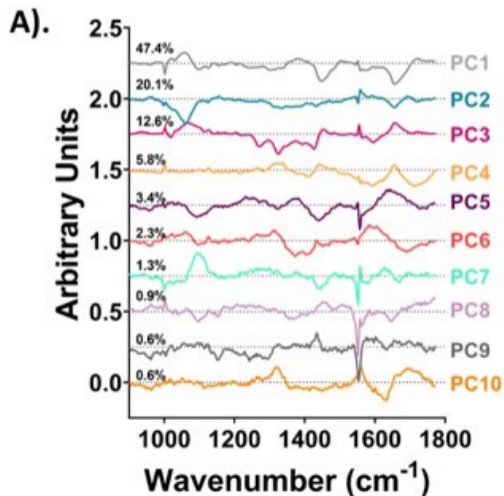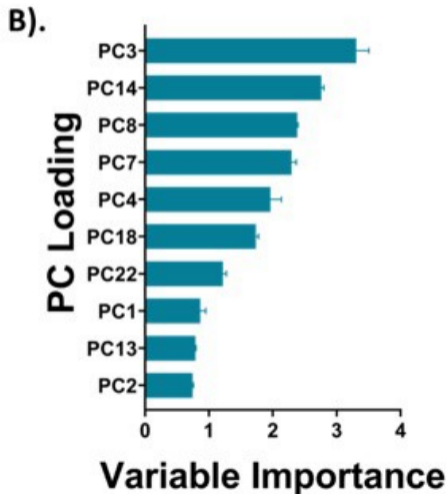

Supplement: AN-148-D3AN00121K-s004 [file AN-148-D3AN00121K-s004.pdf]

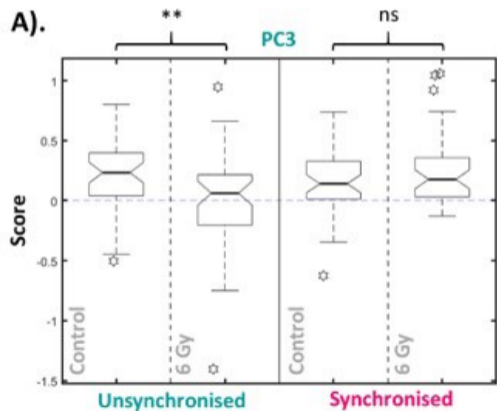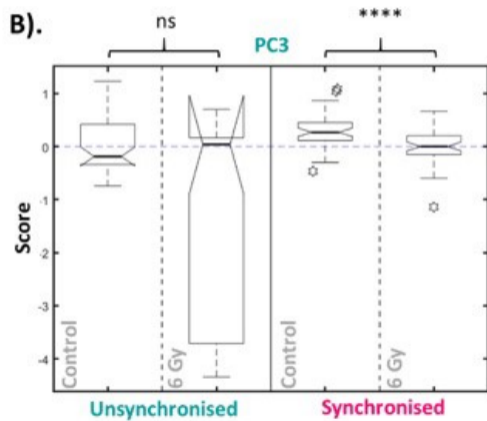

Supplement: AN-148-D3AN00121K-s005 [file AN-148-D3AN00121K-s005.pdf]

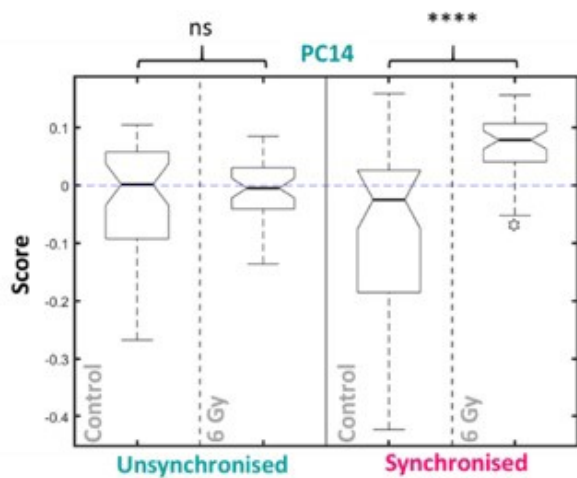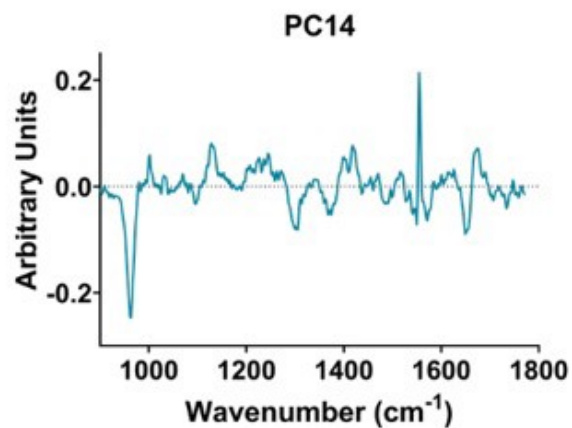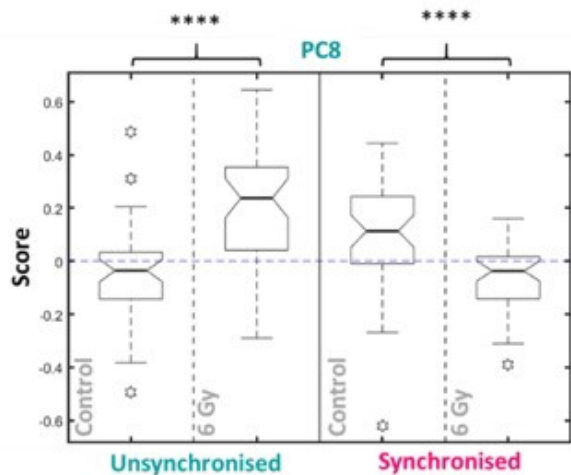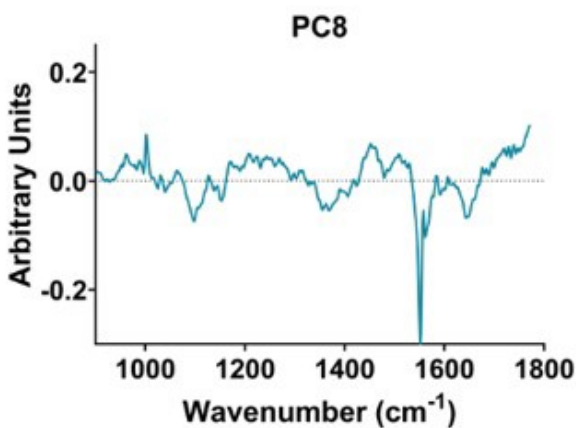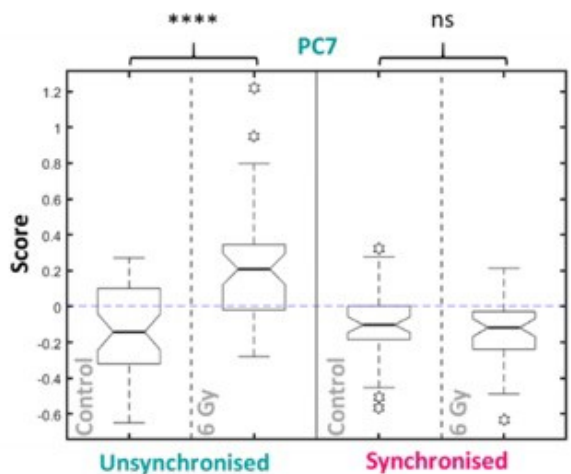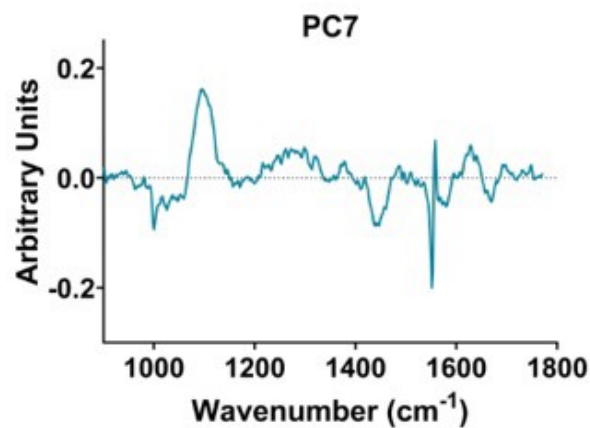

Supplement: AN-148-D3AN00121K-s006 [file AN-148-D3AN00121K-s006.pdf]

1 hour

A).

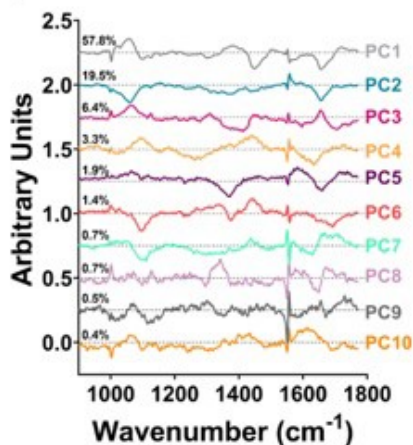

B).

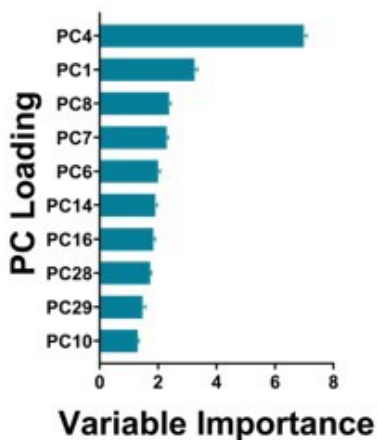

4 hour

A).

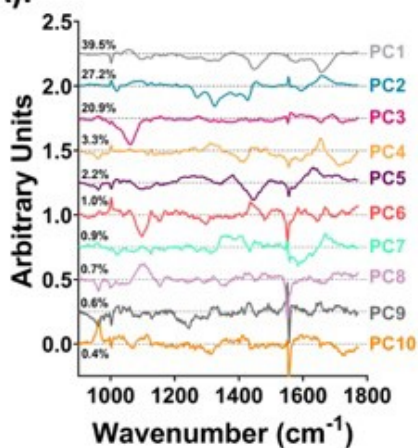

B).

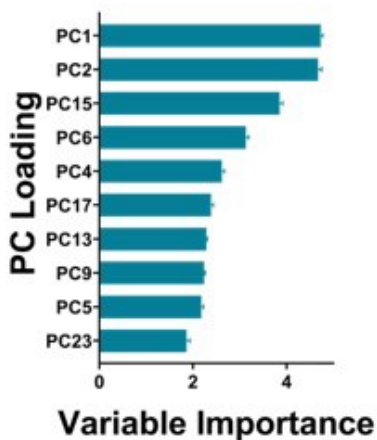

24 hour

A).

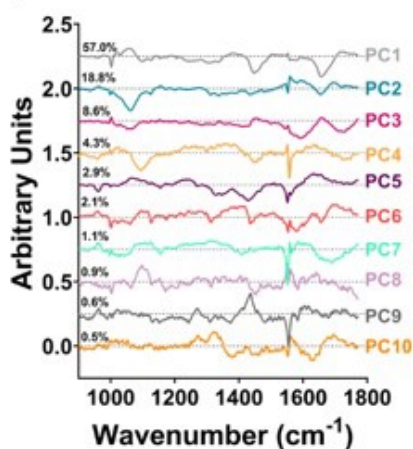

B).

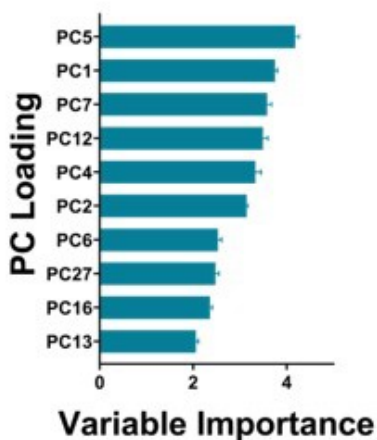

Supplement: AN-148-D3AN00121K-s007 [file AN-148-D3AN00121K-s007.pdf]

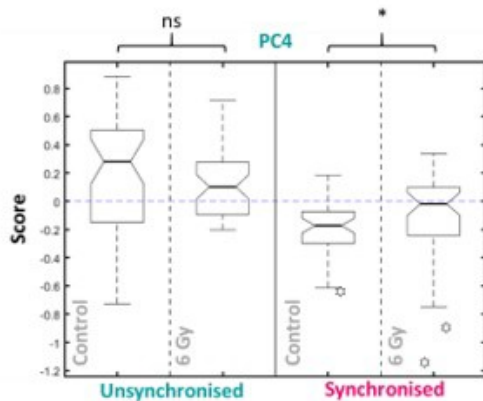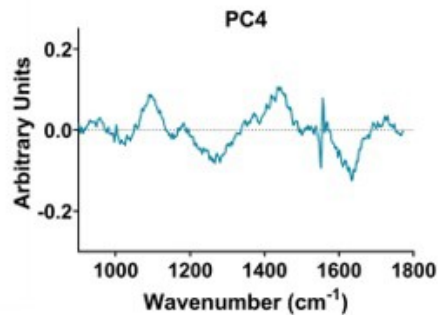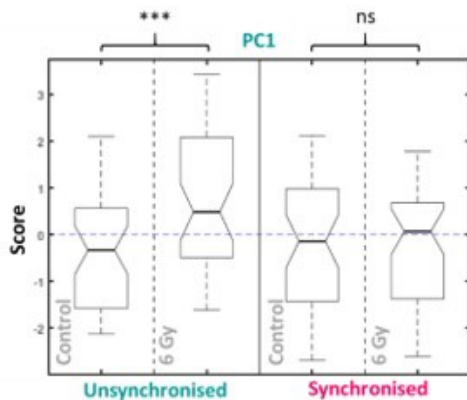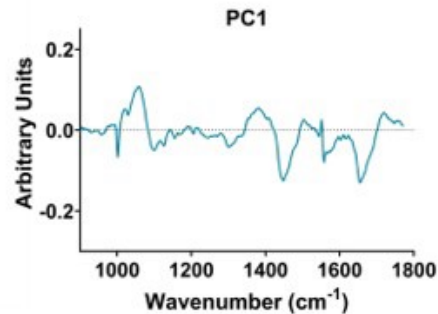

Supplement: AN-148-D3AN00121K-s008 [file AN-148-D3AN00121K-s008.pdf]
