# Supplementary material for: Understanding radiation response and cell cycle variation in brain tumour cells using Raman spectroscopy
Source: Analyst. 2023 May 1;148(11):2594–608. doi: 10.1039/d3an00121k (PMC10228487; doi:10.1039/d3an00121k)
Supplement: AN-148-D3AN00121K-s009 [file AN-148-D3AN00121K-s009.pdf]

Electronic Supplementary Material (ESI) for Analyst.
This journal is © The Royal Society of Chemistry 2023

Supporting Information

Understanding Radiation Response and Cell Cycle Variation in Brain Tumour Cells using Raman Spectroscopy

Iona E Hill,[a] Marie Boyd,[b] Kirsty Milligan,[c] Cerys A Jenkins,[d] Annette Sorensen,[b] Andrew Jirasek,[c] Duncan Graham,[a] Karen Faulds*[a]

[a.] Centre for Molecular Nanometrology, Department of Pure and Applied Chemistry, Technology and Innovation Centre, University of Strathclyde, 99 George Street, Glasgow, G1 1RD, UK
[b.] Strathclyde Institute of Pharmacy and Biomedical Sciences, University of Strathclyde, Glasgow, G1 1XQ, UK
[c.] Department of Physics, The University of British Columbia, Kelowna, Canada
[d.] Swansea University Medical School, Swansea University, Singleton Park, Swansea, SA2 8PP, UK

Materials & Methods

Cell cycle synchronisation: UVW cells were synchronised at the G_1/S boundary by treatment with a double thymidine block. A total of 1×10^5 cells were seeded into 25 cm^3 tissue culture flasks. A stock solution of thymidine (0.1 M) was prepared in phosphate buffered saline (PBS). The stock thymidine solution was added to the cell culture medium to a final concentration of 2 mM. The cells were first treated with thymidine (2 mM) for 18 hours. The treatment was then removed, the cells were washed with PBS and incubated with fresh cell medium for 9 hours. After 9 hours, cells were treated a second time with 2 mM thymidine for 18 hours. After the double thymidine block treatment, cells were washed with PBS and reincubated with fresh medium. UVW cells were synchronised in the S phase using a double thymidine block followed by reincubation with thymidine-free cell culture medium for 3 hours. UVW cells were synchronised at the G_2/M boundary by first treating them with thymidine (2 mM) for 24 hours, removing thymidine treatment and reincubating in thymidine-free cell culture medium for 3 hours. Cells were then treated for 12 hours with nocodazole (100 ng/mL). Finally, UVW cells were synchronised in the G_1 phase of the cell cycle by first synchronising them to the G_2/M boundary, as described above. Following nocodazole treatment, UVW cells were reincubated with nocodazole-free cell culture medium for 5 hours.

Cell cycle analysis: Cell cycle distribution was evaluated by staining the cellular deoxyribonucleic acid (DNA) using propidium iodide and analysed using fluorescence-activated cell sorting (FACS).[1] UVW cells were collected by removing the medium from the sample and washing with PBS. Cells were then harvested with 0.05 % trypsin- ethylenediaminetetraacetic acid (trypsin-EDTA) and once in suspension, 0.05 % trypsin-EDTA was neutralised with cell culture medium. Cells were then pelleted using centrifugation at 311 × g for 5 minutes. Supernatant was removed and the cells were resuspended in 4 ^{o}C, 70 % ethanol (EtOH) to fix. Cells were then stored at -20 ^{o}C until analysis. Prior to analysis, samples were centrifuged at 423 × g for 5 minutes to pellet cells. Supernatant was then removed, and the cells were washed twice in PBS by centrifugation (423 × g, 5 minutes). Fixed cell pellets were then incubated with 50 µg/mL ribonuclease A (RNase A) and 10 µg/mL propidium iodide (PI). RNase was used to degrade the ribonucleic acid (RNA) and therefore prevent staining of intracellular RNA and PI was used to stain cellular DNA. Samples were incubated in a light-free environment at 4 ^{o}C for at least one hour prior to analysis. Samples were transferred into FACS tubes prior to analysis. Cells were analysed by flow cytometry using an Attune NxT Flow Cytometer (ThermoFisher, UK). Three independent experiments were carried out, unless otherwise stated and results were presented as the percentage of cells in each phase of the cell cycle. (mean ± standard deviation).

γ-H2AX assay: DNA damage and repair in UVW cells was quantified by detection of SER139 phosphorylated γ-H2AX which is a histone phosphorylated in response to DNA damage.[2, 3] UVW cells were collected for analysis by detaching them from the 6-well plate with 0.05 % trypsin-EDTA. Cells were centrifuged at 311 × g for 5 minutes to form a pellet and supernatant was then removed. The cell pellet was washed in 1 mL PBS by centrifugation (311 × g, 5 minutes) then PBS was removed. Cells were then fixed by incubation with 1 mL of 4 % (v/v) paraformaldehyde for 15 minutes. Once UVW cells had been fixed, samples were centrifuged (311 × g, 5 minutes) and the 4 % PFA was removed. Fixed UVW cells were then stored in 1 mL of PBS in the fridge (4 ^{o}C) until analysis (maximum of 24 hours). Prior to analysis, samples were centrifuged to form a pellet (423 × g, 5 minutes). Supernatant was removed and cells were permeabilised by resuspension in 0.3 % (v/v) Triton-X-100 for 30 minutes at an approximate density of 2 x 10^6 cells/mL. Following permeabilisation, cells were centrifuged (423 × g, 5 minutes) to form a pellet and excess Triton-X-100 was removed. The cell pellet was then washed with blocking buffer (0.1 % Triton-X-100 and 0.5 % bovine serum albumin (BSA) in PBS) by centrifugation (423 × g, 5 minutes). The cells were then resuspended in blocking buffer and incubated with 100 µg/mL FITC-conjugated anti-phospho-histone H2AX (SER139) antibody (Millipore, UK). Cells were incubated in a light-free environment at 4 ^{o}C for 20 minutes. Following incubation, excess SER139 antibody was removed by centrifugation (423 × g, 5 minutes) and the supernatant was removed. Cells were then resuspended in FACS buffer containing 1 % BSA in PBS. Samples were transferred to FACS tubes and analysed immediately by flow cytometry using an Attune NxT Flow Cytometer (ThermoFisher, UK). Three independent experiments were carried

out, unless otherwise stated, and results were presented as the percentage of cells in each phase of the cell cycle. (mean ± standard deviation).

Data Processing: MATLAB software (R2017a) was used to process the Raman spectra, using in-house MATLAB scripts. This software was used for noise reduction of the spectra using Nonlinear iterative partial least squares (NIPALS) decomposition. The x-axis of the spectra was standardised to the phenylalanine peak at 1004 cm^{-1}. The spectra were baseline corrected using rolling-circle filter (RCF) with radius of 150 units. Quality control was performed on the data which excluded spectra outside the range: mean ± 2.5*SD. Spectra were normalised using an area under the curve (AUC) normalisation. Finally, spectra were truncated to between 900 – 1770 cm^{-1}. Principal component analysis (PCA) was then performed first on all the data combined and then on the data from each timepoint individually. Following PCA, the most important PC score was presented as a Tukey style box plot of the median score. In the Tukey box plot the centre point represented the median PC score value, notches represented the 25th and 75th percentile, whiskers represented the 5th and 95th percentile and stars represented outliers. Random forest (RF) modelling was carried out on PCA scores obtained from the PCA using the open source randomForest package in R software (Version 3.6.1). Data was split into a training (75 % of the data) and a testing (25 % of the data) set. The random forest was constructed using 2000 decision trees and 12 randomly selected input variables were used to split each node. The testing data set was then input to the random forest, which was constructed using the training data set, in order to obtain the classifications of variables. The out-of-bag (OOB) estimate of error was obtained using the training data set. Variable importance was calculated using mean decrease in Gini impurity. ANOVA statistical analysis was carried out using GraphPad Prism 8 software (Version 8.4.2, GraphPad Software Inc., USA). ANOVA was used to generate a p-value of statistical difference between the samples. All tests for cell cycle and γ-H2AX were performed with Bonferroni post-tests at a 95 % confidence interval (CI) and tests for PCA loadings were performed with Wilcoxon rank sum test at 99% CI for PCA loadings. A p-value of less than 0.05 was considered statistically different.

Results & Discussion

Cell cycle synchronisation: Different cell cycle synchronisation methods were initially evaluated to determine a successfully method for synchronising cells to one phase of the cell cycle. Four different methods were used to synchronise the cells to the G_1/S boundary, S phase, G_2/M boundary and early G_1 phase.

Treatment with a double thymidine block was able to successfully synchronise the UVW cells to the G_1/S boundary of the cell cycle.[4] Using this treatment followed by a 3 - 4 hour release also showed promise for synchronisation of cells in the S phase of the cell cycle.[4,5] We also wanted to achieve cell synchronisation at the G_2/M boundary and early G_1 phase of the cell cycle to study four different phases of the cell cycle.

For G_2/M synchronisation, UVW cells were treated for 24 hours with 2 mM thymidine followed by a 3-hour incubation with drug-free medium, cells were then treated for 12 hours with 100 ng/ml nocodazole.[4,5] At this stage cells were collected with trypsin-EDTA and fixed for analysis. Previous work has used a mitotic shake-off method to separate mitotic cells following treatment. However, in this study collecting samples by mitotic shake-off led to a very low cell yield compared to the other samples (G_1/S phase and S phase) collected. The low collection yield rendered the sample impractical for analysis using Raman spectroscopy.

Early G_1 phase synchronisation was achieved using the same method as the G_2/M boundary cells described above, however after cells were collected with trypsin, they were reincubated with drug-free medium for 5 hours to allow cells to adhere to the imaging substrate and progress through mitosis to early G_1 phase.[5]

The cell cycle distribution of all treated samples is shown in Figure S1. The FACS of the untreated control showed that 1.2 % of cells occupied the sub-G_1 phase of the cell cycle, 52.5 % occupied the G_0/G_1 phase of the cell cycle, 11.8 % occupied the S phase of the cell cycle and 34.5 % of cells occupied the G_2/M phase of the cell cycle (Figure S1-A).

The results of G_1/S boundary are shown in Figure S1-B. This achieved a significant increase in G_0/G_1 population ($p < 0.0005$) and a significant decrease in G_2/M population ($p < 0.0001$) compared to the untreated control cells. The analysis showed there was 71.0 % of the cell population at the G_1/S boundary of the cells cycle. This demonstrated that the majority of cells were synchronised, however 3.1 % still populated sub-G_1 phase, 11.0 % in S phase and 14.9 % in G_2/M phase.

S phase synchronisation was achieved using a double thymidine block treatment followed by a 3-hour incubation with drug-free medium and the results are shown in Figure S1-C. The results showed that 5.0 % of cells occupied the sub-G_1 phase, 37.2 % in the G_0/G_1 phase, 40.6 % in the S phase and 17.2 % in the G_2/M phase. Compared to the untreated control cells, these results showed a significant increase in S phase population ($p < 0.0001$), and significant decrease in G_2/M phase ($p < 0.0005$) and G_0/G_1 phase ($p < 0.005$) populations. Although the majority of cells occupied the S phase, a large population were still present in the G_0/G_1 phase of the cell cycle. This could suggest that the cells had not released as quickly from the treatment as previous experiments which may indicate that a longer treatment release was required.

The cell cycle distribution of cells synchronised to the G_2/M boundary is shown in Figure S1-D. The FACS analysis showed that 86.6 % of cells occupied the G_2/M phase of the cell cycle following synchronisation which was a significant increase in G_2/M population ($p < 0.0001$) compared to the untreated control cells. In the other regions of the cell cycle, 4.2 % of cells occupied the sub-G_1 phase, 3.9 % the G_0/G_1 phase and 5.1 % the S phase. Therefore, the synchronisation using this method was deemed to be a suitable methodology to be taken forward for further experiments.

Finally, the cell cycle analysis of cells synchronised in early G_1 phase are shown in Figure S1-E. The results showed that 60.3 % of cells occupied the G_0/G_1 phase of the cell cycle. This may suggest that the majority of cells had been successfully synchronised in the early G_1 phase of the cell cycle, however the cell cycle distribution was not significantly different compared to the untreated control cells ($p > 0.05$). The results showed that in this sample, 5.6 % of cells occupied the sub-G_1 phase, 7.6 % in the S phase and 26.5 % in the G_2/M phase. There were still some cells in the G_2/M phase of the cell cycle which may suggest that the 5-hour re-incubation was not sufficient for all cells to transition into the G_1 phase.

Overall, all methods of synchronisation led to the majority of cells occupying the desired phase of the cell cycle. However, the precision of the synchronisation did vary between methods. For further experiments the double thymidine block method was chosen to synchronise cells to the G_1/S boundary of the cell cycle as this showed successful results, with results similar to what has been reported previously.[5,6]

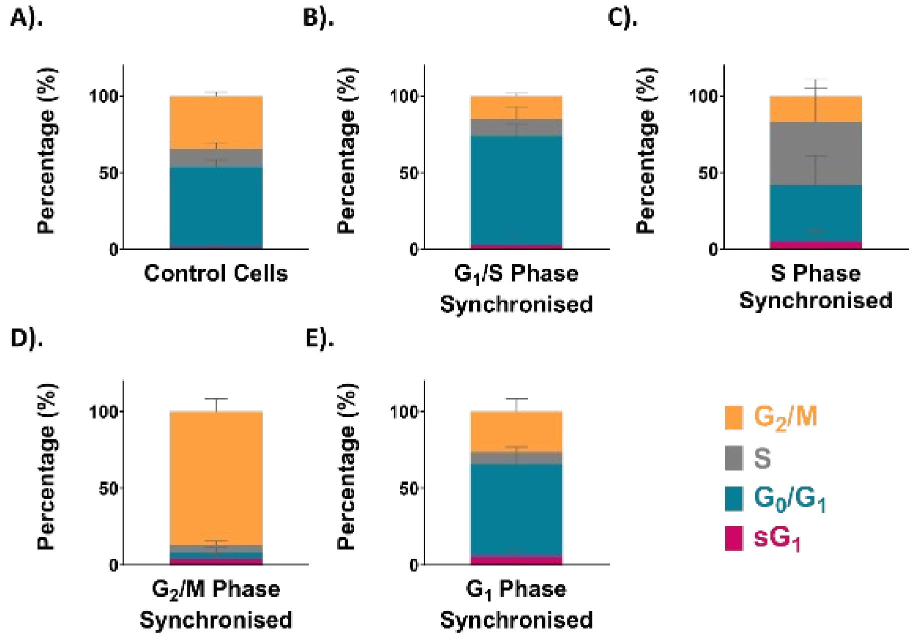

Figure S1 FACS analysis of UVW cells synchronised to different phases of the cell cycle. A). Unsynchronised control cells, B). G_1/S boundary, C). S phase, D). G_2/M boundary, and E). Early G_1 phase of the cell cycle. FACS analysis results of the percentage population of cells occupying each position in the cell cycle. Bars represent percentage of cells in sG1 phase (pink), G0/G1 phase (blue), S phase (grey) and G2/M phase (yellow). Data represents the average results from at least three independent replicates (mean + standard deviation). Two-way ANOVA compared the mean cell cycle phase population in each treated cell population compared to the control untreated cells. Statistical analysis was performed using two-way ANOVA with Bonferroni post-tests at 95 % confidence interval ($p > 0.05$ = ns, $p < 0.05$ = *, $p < 0.005$ = **, $p < 0.0005$ = *** and $p < 0.0001$ = ****).

Raman analysis: A large peak at 1555 cm^{-1} was observed in the sample spectra which may be assigned to tryptophan/amide I. However, the appearance of this peak was very sharp and this feature was dominant in the Raman spectrum. These characteristics would be unusual in a cell spectrum. Raman analysis of a clean CaF_2 showed that this feature was present indicating that it was a result of CaF_2 or instrumental background (Figure S2). For this reason, this feature was not included as a contributing factor in radiation response.

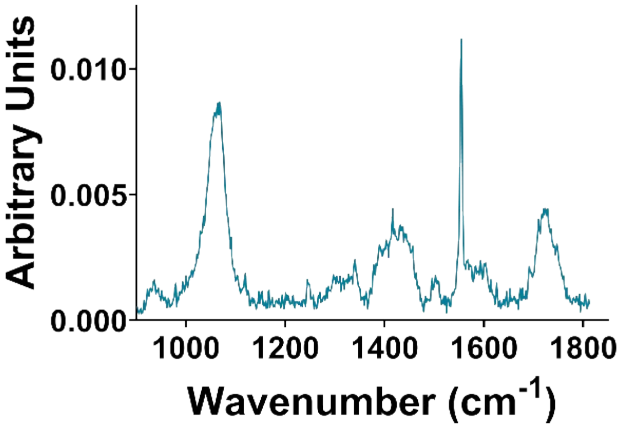

Figure S2 Background off-sample Raman spectrum of blank CaF2 coverslip representing average spectrum of five separate Raman maps of clean CaF2 window. Spectrum was obtained using a Renishaw InVia Raman microscope using a 532 nm laser wavelength, 50x objective, 1.0 second acquisition time, 100 % laser power (50 mW at source, 21 mW at sample) and 1 µm step size. Raman spectra were processed by cosmic ray removal, noise reduction, baseline correction, and area under the curve (AUC) normalisation.

Data analysis of Raman spectra: Principal component analysis (PCA) was used to compare the average Raman spectra from each sample group first by including all the data together then for the timepoints separately.

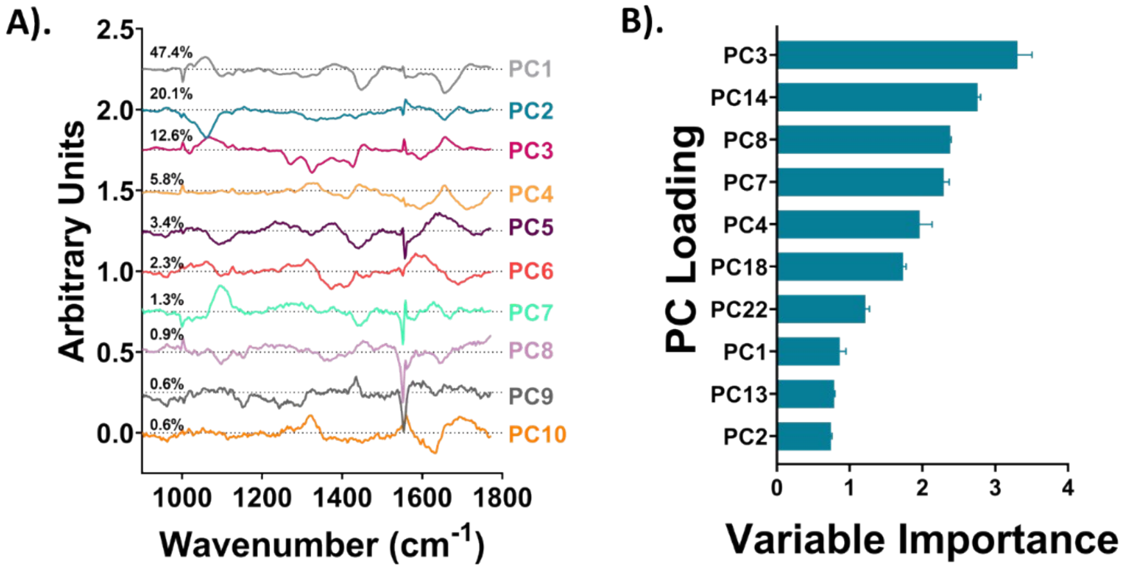

Figure S3 PCA loadings and RF classification analysis. A). PC loadings (PC1 – PC10) from PCA comparing average cell spectra from each sample condition. Showing percentage variance explained for each PC loading (%). B). Measure of variable importance (arbitrary units) of PC loading scores in RF model displaying only the 10 most important PC loadings. RF was carried out on the average spectra of each cell in all sample groups. The variable importance was obtained by the average of five RF models and the bars represent average and standard deviation for each PC loading. RF model consisted of 2000 trees and 12 variables were randomly selected to split each node. (The data shows the first 15 PC loadings with highest variable importance).

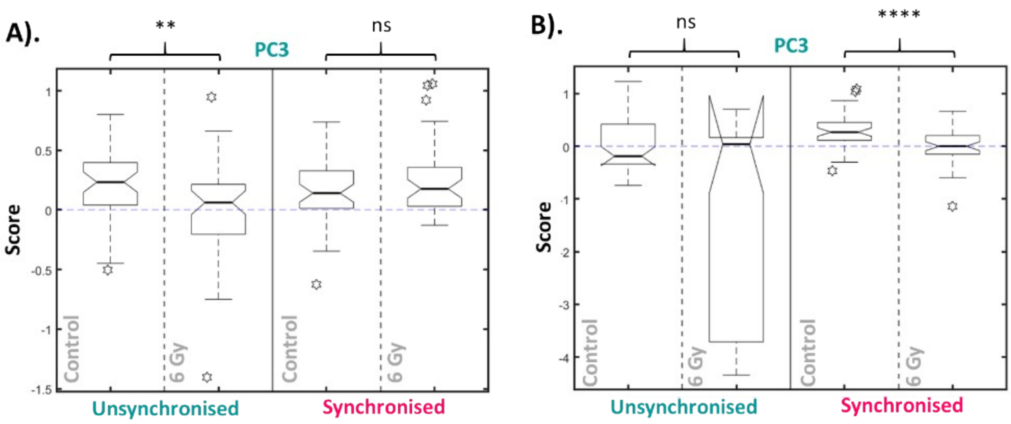

Figure S4 Principal component analysis (PCA) of average Raman spectra showing box plot of PC3 median loading scores at the A). 1 hour time point, and B). 4 hour timepoint. Box plot compares control cells and 6 Gy irradiated cells for unsynchronised UVW cells (blue) and synchronised UVW cells (pink). Centre point of box represents median value, notches represent the 25th and 75th percentile, whiskers represent the 5th and 95th percentile and stars represent outliers. B). PC3 loading from PCA comparing control and 6 Gy samples for all timepoints following 6 Gy XBR exposure for unsynchronised and synchronised UVW cells. Statistical analysis was performed using a two-way ANOVA with Wilcoxon rank sum test at 99% confidence interval ($p > 0.05$ = ns (not significant), $p<0.01$ = ** and $p < 0.0001$ = ****).

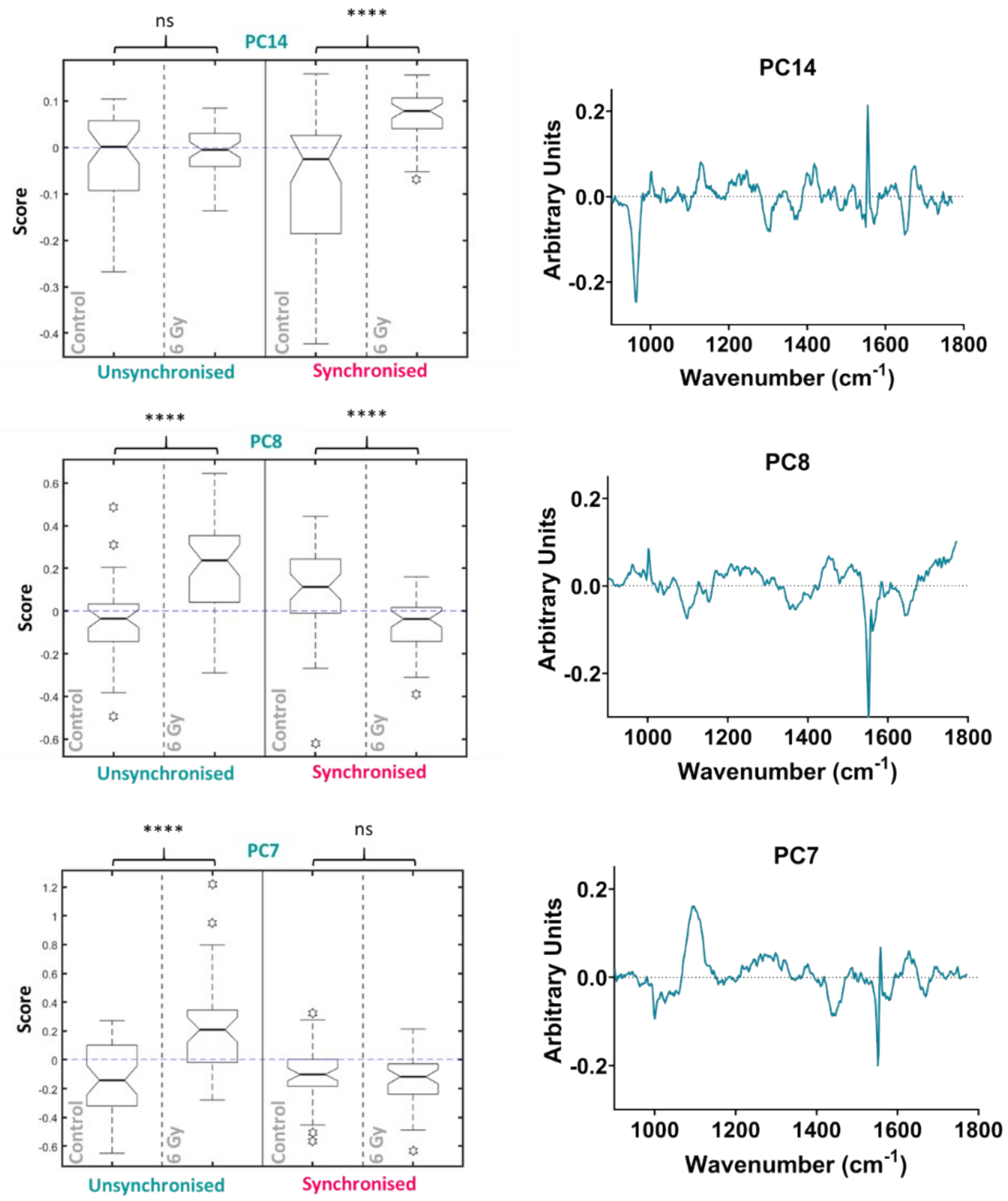

Figure S5 Principal component analysis (PCA) of average Raman spectra for the 24 hour timepoint showing the box plots and PC loadings for PC14, PC8 and PC7. Box plot compares control cells and 6 Gy irradiated cells for unsynchronised UVW cells (blue) and synchronised UVW cells (pink). Centre point of box represents median value, notches represent the 25th and 75th percentile, whiskers represent the 5th and 95th percentile and stars represent outliers. Statistical analysis was performed using a two-way ANOVA with Wilcoxon rank sum test at 99% confidence interval ($p > 0.05$ = ns (not significant and $p < 0.0001$ = ****).

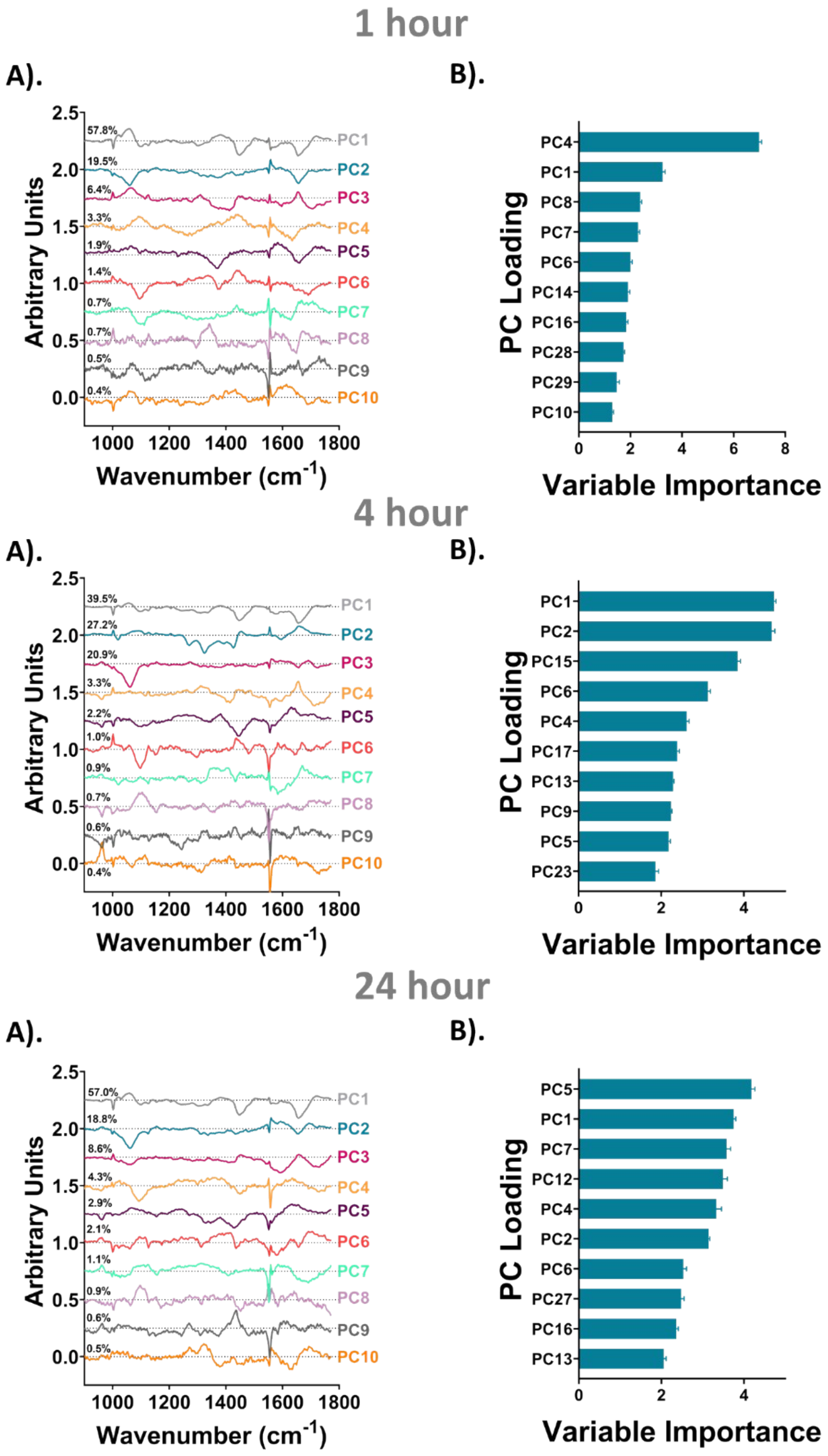

Figure S6 PCA loadings and RF classification analysis for 1 hour, 4 hour and 24 hour timepoints individually. A). PC loadings (PC1 – PC10) from PCA comparing average cell spectra from each sample condition. Showing percentage variance explained for each PC loading (%). B). Measure of variable importance (arbitrary units) of PC loading scores in RF model displaying only the 10 most important PC loadings. RF was carried out on the average spectra of each cell in all sample groups. The variable importance was obtained by the average of five RF models and the bars represent average and standard deviation for each PC loading. RF model consisted of 2000 trees and 12 variables were randomly selected to split each node. (The data shows the first 15 PC loadings with highest variable importance).

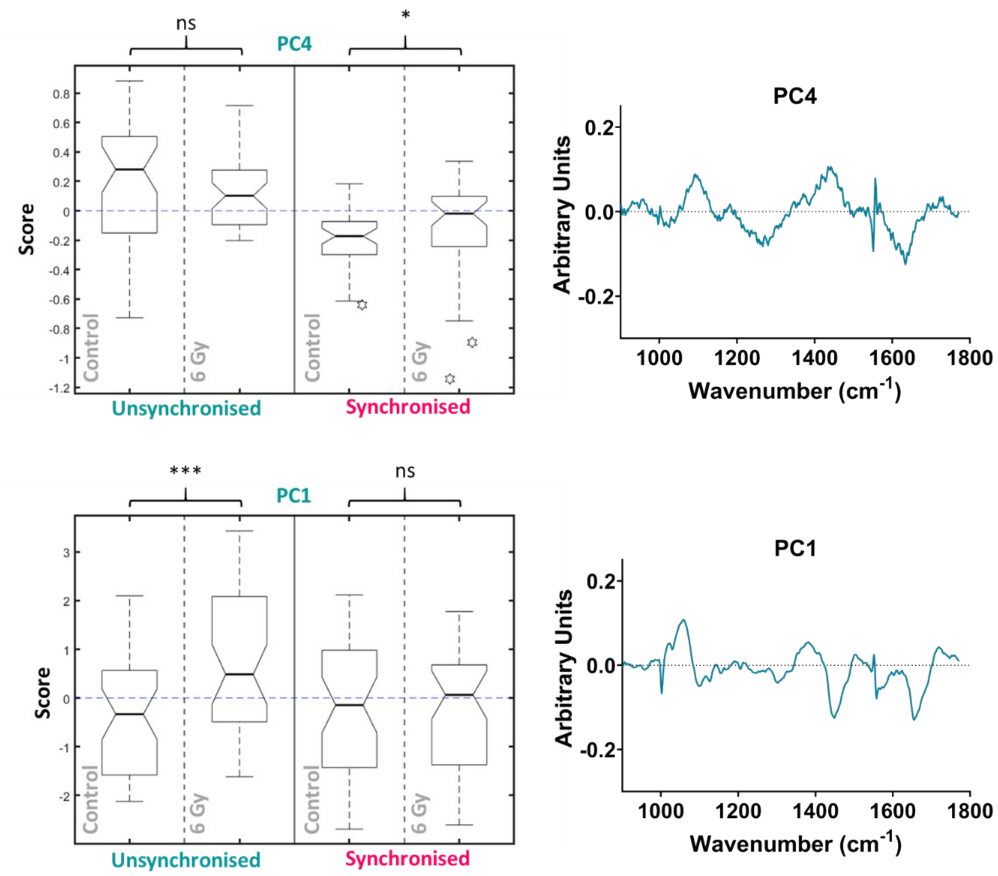

Figure S7 Principal component analysis (PCA) of average Raman spectra for the 1 hour timepoint showing the box plots and PC loadings for PC4 and PC1. Box plot compares control cells and 6 Gy irradiated cells for unsynchronised UVW cells (blue) and synchronised UVW cells (pink). Centre point of box represents median value, notches represent the 25th and 75th percentile, whiskers represent the 5th and 95th percentile and stars represent outliers.

References

1 Z. Darzynkiewicz, X. Huang and H. Zhao, Analysis of cellular DNA content by flow cytometry, Current Protocols in Immunology, 2017, 2017, 5.7.1-5.7.20.

2 S. Burma, B. P. Chen, M. Murphy, A. Kurimasa and D. J. Chen, ATM Phosphorylates Histone H2AX in Response to DNA Double-strand Breaks, Journal of Biological Chemistry, 2001, 276, 42462–42467.

3 H. Zhao, X. Huang, H. D. Halicka and Z. Darzynkiewicz, Detection of Histone H2AX Phosphorylation on Ser-139 as an Indicator of DNA Damage, Current Protocols in Cytometry, 2019, 89, e55.

4 G. Chen and X. Deng, Cell Synchronization by Double Thymidine Block, Bio-protocol, 2018, 8, e2994.

5 A. G. Brolo, A. Jirasek, J. Lum, Q. Matthews and X. Duan, Variability in Raman Spectra of Single Human Tumor Cells Cultured in Vitro: Correlation with Cell Cycle and Culture Confluency, Applied Spectroscopy, Vol. 64, Issue 8, pp. 871-887, 2010, 64, 871–887.

6 R. J. Swain, G. Jell and M. M. Stevens, Non-invasive analysis of cell cycle dynamics in single living cells with Raman micro-spectroscopy, Journal of cellular biochemistry, 2008, 104, 1427–1438.
